# C2C12‐Derived ApoVs Promote Skeletal Muscle Development and Ameliorate Age‐Related Muscle Loss Through Igf1r/PI3K/AKT/mTOR Pathway

**DOI:** 10.1002/jcsm.70159

**Published:** 2025-12-04

**Authors:** Aiwen Jiang, Yi Liu, Luyao Wang, Haifei Wang, Shenglong Wu, Wenbin Bao

**Affiliations:** ^1^ Key Laboratory for Animal Genetics, Breeding, Reproduction and Molecular Design, College of Animal Science and Technology Yangzhou University Yangzhou China; ^2^ Joint International Research Laboratory of Agriculture & Agri‐Product Safety Yangzhou University Yangzhou China

**Keywords:** apoptosis, apoVs, Igf1r, myogenic differentiation

## Abstract

**Background:**

Apoptosis coincides with the differentiation of skeletal myoblasts, and numerous studies have shown that the apoptotic activity is required for myogenic differentiation. Although the role of apoptosis in skeletal muscle differentiation has been well documented, its mechanism is largely unknown.

**Methods:**

Apoptotic extracellular vesicles (apoVs) were extracted from differentiated C2C12 cells or STS‐treated undifferentiated C2C12 myoblasts. C2C12 myoblasts, 8‐week‐old male mice or 15‐month‐old male mice were used as in vitro and in vivo models, respectively. These models were treated with C2C12‐derived apoVs to explore the biological function and mechanism of apoVs in myogenic differentiation, skeletal muscle development and aging.

**Results:**

Proteomic analysis revealed that inhibition of apoptotic activity by Z‐VAD‐FMK (ZVAD) affected extracellular components. Using immunofluorescence staining, western blotting and transmission electron microscopy analysis, our results demonstrated the generation of apoVs during myogenic differentiation. C2C12‐derived apoVs exhibited a typical double‐membrane spherical structure, phosphatidylserine exposure and were highly positive for the general apoV markers cleaved caspase 3 (CASP3), Alix and TSG101. Inhibition of apoptotic activity significantly reduced (*p* = 0.0029) the protein level of myosin heavy chain (1.05 in the Con group vs. 0.38 in the ZVAD group) accompanied by a marked decrease (*p* < 0.0001) in apoV production (5.91e+10 ± 8.93e+09 in the Con group vs. 1.77e+10 ± 1.36e+09 in the ZVAD group). Proteomic analysis of apoVs suggested that C2C12‐derived apoVs contain multiple pro‐differentiation proteins, including insulin‐like growth factor 1 receptor (Igf1r). ApoVs could be taken up by recipient cells and subsequently rescued the impaired C2C12 differentiation induced by ZVAD (*p* < 0.0001) and promoted the normal myogenic differentiation process (*p* = 0.0081) by carrying Igf1r and promoting PI3K/AKT/mTOR activation. Knockdown of Igf1r or inhibition of PI3K activation diminished the positive role of apoVs. In addition, apoV treatment promoted skeletal muscle development in 8‐week‐old male mice (*n* = 6, Cohen's d = 1.993, power = 0.874) and relieved age‐related muscle loss (*n* = 6, Cohen's d = 3.97, power = 0.999).

**Conclusions:**

In summary, this study demonstrates the generation of apoVs during myogenic differentiation. The apoVs derived from skeletal muscle cells promote skeletal muscle cell differentiation and delay age‐related muscle loss. These results provide a theoretical basis for elucidating the mechanism of skeletal muscle development and treating skeletal muscle‐related diseases.

## 1 Introduction

As the largest organ in the body, skeletal muscle occupies over 40% of body weight [1]. It is not only responsible for body movement but also plays important roles in metabolic regulation and temperature maintenance [2]. The disorders of skeletal muscle development are closely associated with aging, type 2 diabetes, cancer and muscle‐related diseases, such as amyotrophic lateral sclerosis (ALS) and Duchenne muscular dystrophy (DMD) [3, 4, 5]. Therefore, in‐depth understanding of skeletal muscle development mechanisms is helpful to maintain skeletal muscle function and alleviate the progression of aging and diseases.

Skeletal muscle development is a complex, multistage and tightly regulated biological process that continues from the embryo to adulthood. Before birth, myoblast cells originating from Pax3^+^/Pax7^+^ muscle stem cells proliferate rapidly to expand the number of cells [6]; then, myoblasts exit the cell cycle and start the myogenic differentiation process under the regulation of myogenic regulatory factors (MRFs) to form myocytes [7]. Myocytes fuse with each other to form a multinucleated myotube, which further matures and expresses sarcomere‐related proteins, such as skeletal‐α‐actin and myosin, to possess the contractile function [8]. In mammals, the number of muscle fibres is basically fixed in the embryonic period, and postnatal muscle development mainly depends on the hypertrophy of muscle fibres, which reflects skeletal muscle growth via protein synthesis and catabolism [9]. Key signalling pathways that promote muscle growth are clear, such as insulin‐like growth factor 1 (IGF‐1), PI3K‐AKT‐mTOR, Ras‐MAPK‐ERK and Wnt pathways [10, 11, 12, 13]. Biological events, including cell cycle regulation, DNA damage repair, cytoskeleton remodelling and cell apoptosis, play key roles in myogenesis by regulating the myogenic differentiation process [14, 15, 16, 17]. More importantly, skeletal muscle has the ability to regenerate after injury; muscle stem cells in adults, named muscle satellite cells, can be activated by stimuli; cytokines released by apoptotic cells might regulate the behaviour of muscle satellite cells by controlling macrophage activation [18].

Apoptosis is an active process of programmed cell death, which is characterised by DNA fragmentation, caspase activation and phosphatidylserine (PtdSer) exposure [19]. Researchers found that apoptosis coincides with the differentiation of skeletal myoblasts [20]. In addition, apoptosis‐associated events such as nuclear degradation, cytoskeletal reorganisation, caspase activation and PtdSer exposure are necessary and beneficial for myogenic differentiation and skeletal muscle regeneration [15, 16, 21, 22]. Receptors of PtdSer, including stabilin‐2 and BAI1, modulates myoblast fusion [23, 24], while the deficiency of Tmem30a, a PS flippase β‐subunit, results in delayed skeletal muscle regeneration [25]. Of note, multiple caspase enzymes, such as caspase‐3, caspase‐2 and caspase‐9, are reported to control myotube formation by inducing DNA strand breaks or activating downstream signalling pathways [26, 27, 28]; the inhibition of caspase activity by Z‐VAD‐FMK (ZVAD), a pan‐caspase inhibitor, blocks myogenic differentiation, while the addition of apoptotic myoblasts promotes myoblast fusion [24]. Although the role of apoptosis in skeletal muscle differentiation has been well documented, the underlying mechanism is largely unknown.

Here, we report that the apoptotic cells generated during myogenic differentiation can release apoptotic extracellular vesicles (apoVs), and this process relies on caspase activity. In addition to expressing the general apoV markers cleaved caspase 3 (CASP3), TSG101, Alix and exhibiting PtdSer exposure, C2C12‐derived apoVs contain multiple pro‐differentiation proteins, including insulin‐like growth factor 1 receptor (Igf1r). These apoVs rescue caspase inhibition–induced impairment of myogenic differentiation, and promote skeletal muscle hypertrophy, and alleviate age‐related muscle loss through Igf1r‐mediated PI3K/AKT/mTOR activation.

## 2 Materials and Methods

### 2.1 Reagents

ZVAD (50 μM, HY‐16658B), staurosporine (STS, 500 nM, HY‐15141), PKH26 (100 μM, HY‐D1451), PKH67 (100 μM, HY‐D1421) and LY294002 (10 μM, HY‐10108) were purchased from MedChemExpress (MCE, New Jersey, United States).

### 2.2 Cell Culture and Treatment

C2C12 is a well‐established murine skeletal myoblast cell line widely used in studies on skeletal muscle differentiation, proliferation and myogenic regulatory mechanisms. The C2C12 myoblasts were purchased from Stem Cell Bank of Chinese Academy of Sciences (Shanghai, China) and seeded into T25 or T75 flasks. These cells were maintained in growth medium (GM), which comprised Dulbecco's Modified Eagle's Medium (DMEM; Hyclone Laboratories, Logan, UT, USA) containing 10% (v/v) foetal bovine serum (FBS) (Sigma‐Aldrich, St. Louis, MO, USA), and differentiated in differentiation medium (DM), which comprised DMEM containing 2% (v/v) horse serum (HS) (Sigma‐Aldrich, St. Louis, MO, United States). Undifferentiated C2C12 cells were treated with 50 μM ZVAD for 6 h or transfected with CASP3 siRNAs for 24 h; then, the GM was replaced with DM, and the cells were cultured for 72 h to explore the effect of apoptotic activity on myogenic differentiation. Igf1r siRNAs were transfected into undifferentiated C2C12 myoblasts for 24 h, and then, the differentiation process was induced for 72 h. ApoVs generated from Igf1r siRNA‐transfected C2C12 cells were named apoVs^siIgf1r^. Both CASP3 and Igf1r siRNAs (Table S1) were purchased from GenePharma (Shanghai, China). Transfection was performed via the Lipofectamine 3000 reagent (Invitrogen, Carlsbad, CA, United States) according to the manufacturer's instructions.

### 2.3 Animals

The C57BL/6J male mice at 8 weeks of age were used to detect the effect of apoVs on skeletal muscle hypertrophy. 100 μL of PKH26 or PKH67‐labelled apoVs at a concentration of 2 μg/g were injected into the tibialis anterior (TA) muscle. In the control group, an equal volume of PBS was injected. After continuous injection once a week for 8 weeks, the TA muscle was isolated for detection. Male mice aged from 3 to 21 months were used to measure muscle grip strength. Subsequently, 15‐month‐old mice were selected for the injection of apoVs. TA muscles were collected at 17 months old.

### 2.4 Protein Extraction and Western Blot Analysis

Total protein was extracted using a RIPA buffer (Applygen, Beijing, China), and western blot analysis was conducted as previously described [14]. The antibody information used in this experiment is shown in Table S2.

### 2.5 Cell Apoptosis Analysis

C2C12 cells grown in GM or DM were trypsinised and washed twice with phosphate‐buffered saline (PBS, HyClone, Logan, UT, United States). Then, cells were resuspended in 500 μL binding buffer and incubated with 5 μL Annexin V‐FITC and 5 μL propidium iodide (PI) for 5 min in the dark. Samples were assessed on a FACS Calibur flow cytometer (Becton Dickinson, San Diego, CA, United States). Data were analysed using the Flowjo software. A total of 10 000 cells were analysed per sample.

### 2.6 Cell Cycle Analysis

C2C12 cells at different differentiation times or treated with ZVAD were trypsinised, washed with PBS and fixed in precooled 75% (v/v) ethanol overnight at 4°C. The following day, ethanol was removed after centrifugation, and cells were resuspended in 400 μL PBS and then incubated with 20 μL RNase A solution for 30 min at 37°C. Finally, cells were incubated with 400 μL PI staining solution for 60 min at 4°C in the dark. The RNaseA and PI staining solutions were obtained from Cell Cycle Assay Kit (Vazyme, Nanjing, China). Samples were assessed on a FACS Calibur flow cytometer (Becton Dickinson, Franklin Lakes, NJ, United States). Data were analysed using the ModFit32 software (Verity Software House, Topsham, ME, United States). A total of 20 000 cells were analysed per sample.

### 2.7 Apoptosis Induction of C2C12 Cells

Two methods were used to induce apoptosis in C2C12 cells. The first method involved inducing the differentiation of C2C12 cells in DM for 24 h, after which apoptotic cells [24] or apoVs generated during the differentiation process were collected and named DM.cells or DM.apoVs, respectively. The second method entailed treating undifferentiated C2C12 cells with 500 nM STS; 12 h later, apoptotic cells or apoVs induced by STS were collected and named STS.cells or STS.apoVs, respectively. To investigate the impact of apoptotic activity on apoV production during differentiation, undifferentiated C2C12 cells were treated with 50 μM ZVAD for 6 h; then, the GM was replaced with DM for 24 h of culture. Subsequently, DM.apoVs were collected to detect the protein concentration of apoVs or to determine the particle content using nanoparticle tracking analysis (NTA). In six‐well plates, apoptotic cells (DM.cells or STS.cells) were used at 1 × 10^3^ cells/well, and apoVs (DM.apoVs or STS.apoVs) were used at 20 ng/μL.

### 2.8 Collection and Identification of C2C12‐Derived ApoVs

C2C12‐derived apoVs were collected from differentiated C2C12 cells (DM.apoVs) or STS‐treated undifferentiated C2C12 myoblasts (STS.apoVs). Then, the characteristics of apoVs were identified. Western blot analysis for Cleaved CASP3, Alix and TSG101 was used for detecting apoptotic markers. The non‐vesicle control group was defined as the 100 000 × g supernatant after apoV depletion. NTA was performed to detect size distribution, particle concentration and the ratio of particle to protein (particles/μg); transmission electron microscope (TEM) was used to observe the morphology and size of C2C12‐derived apoVs. The specific experimental procedures were referred to in the published articles [29].

### 2.9 Proteomic Analysis

Protein lysates from C2C12 myocytes treated with ZVAD or its negative control were collected to analyse the changes in the protein profile induced by ZVAD. Additionally, DM.apoVs, STS.apoVs and the parental C2C12 cells were collected to obtain the protein information within apoVs. All samples were analysed by Shanghai Applied Protein Technology (Shanghai, China). Samples were subjected to LC–MS/MS on a timsTOF Pro (ABSCIEX, United States) equipped with a NanoSpray III ion source. Raw data were processed using MaxQuant 1.5.3.30 with the Andromeda search engine, and proteins were identified against the Uniprot database with FDR ≤ 0.01 for both peptides and proteins. Quantification was performed using default MaxQuant parameters. Differentially expressed proteins (DEPs) were filtered by fold change > 1.5 and adjusted *p*‐value (*Q* value) < 0.01, followed by functional enrichment analysis using Gene Ontology (GO) and Kyoto Encyclopedia of Genes and Genomes (KEGG) databases.

### 2.10 Immunofluorescence Assay

Immunofluorescence assay was performed as previously described to determine MyHC positive cells and calculate fusion index [14].

### 2.11 Statistical Analysis

Statistical analyses were performed using the Prism 6 software (GraphPad Software, La Jolla, CA, United States). Results are expressed as means ± standard deviation (SD). The number of biological replicates is indicated by dots in the statistical graphs. Comparisons between two groups were analysed using the two‐tailed unpaired Student's *t*‐test. For data involving three or more groups, one‐way analysis of variance (ANOVA) combined with Tukey's post hoc test was applied. *p* < 0.05 was considered significant. Post hoc statistical power analysis was performed using G. Power (v. 3.1.9.7) to evaluate the treatment effect of apoVs.

## 3 Results

### 3.1 The Extracellular Component Is Affected by Caspase Inhibition in C2C12 Myoblasts

**FIGURE 1:**
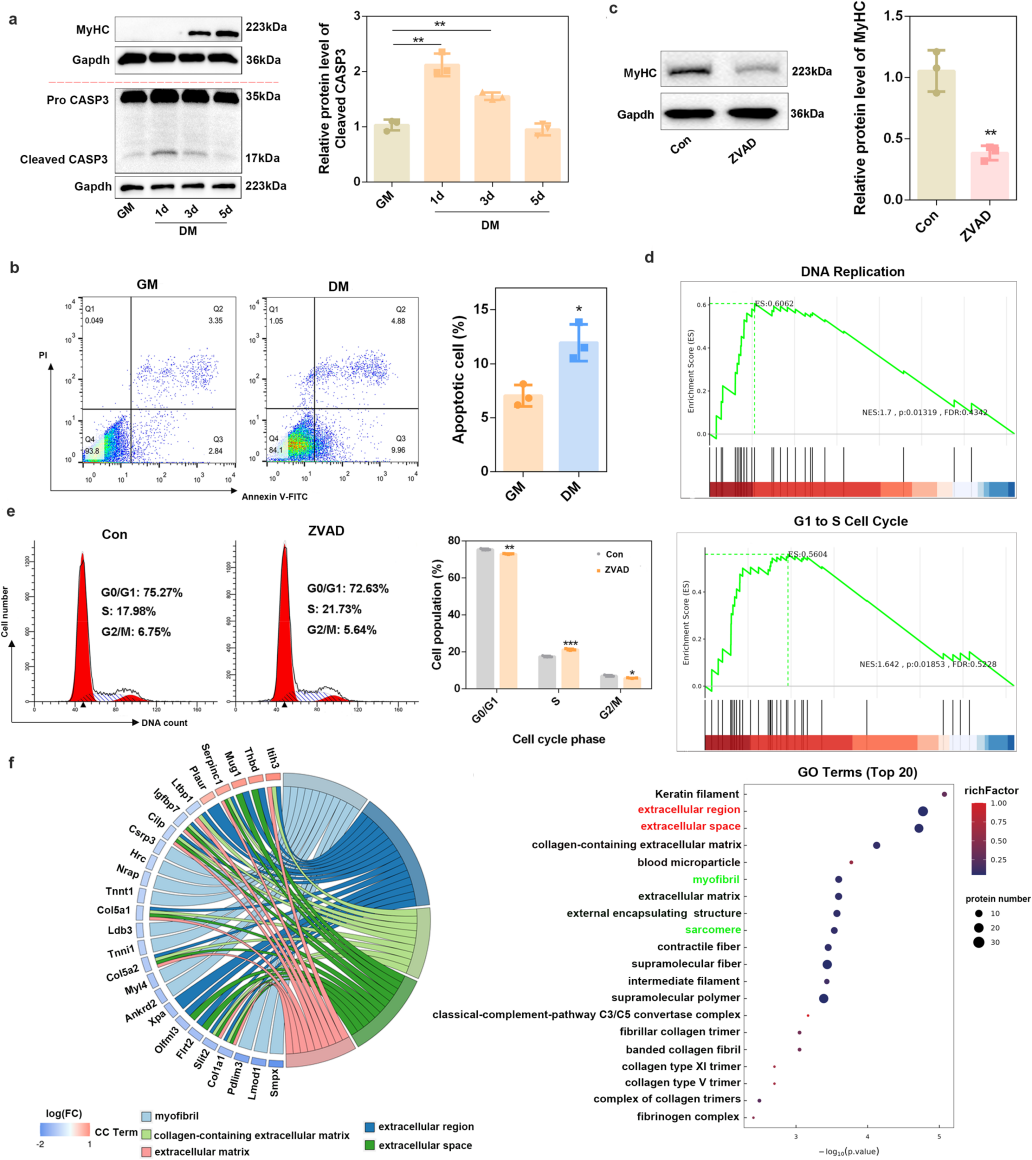
The extracellular component is affected by caspase inhibition in C2C12 myoblasts. (a) Expression pattern of MyHC and caspase3 (CASP3) during C2C12 differentiation was determined by western blot analysis. Gapdh was used as the loading control, and protein signal intensities were analysed using the ImageJ software (*n* = 3). (b) The apoptotic rate of C2C12 cells 1 day after differentiation was determined by flow cytometry (FACS). The percentage of apoptotic cells was quantified by Flowjo after the indicated treatments (*n* = 3). (c) C2C12 myoblasts pre‐treated with 50 μM ZVAD were induced to differentiate for 3 days, and then, the protein levels of MyHC were determined by western blot analysis. Gapdh was used as the loading control, and protein signal intensities were analysed using the ImageJ software (*n* = 3). (d) Gene set enrichment analysis (GSEA) was performed using the differential expressed proteins (DEPs) between the Con group and the ZVAD group. (e) C2C12 myoblasts pre‐treated with 50 μM ZVAD were induced to differentiate for 12 h, and then, cell cycle analysis was performed. Data were analysed using the ModFit32 software (*n* = 3). (f) Chord Diagram and Top 20 terms of Cellular Component (CC) in GO enrichment analysis for the DEPs between the Con group and the ZVAD group. *p < 0.05; **p < 0.01; ***p < 0.001.

C2C12 cells were induced to differentiate for 5 day after replacing GM with DM. The protein level of myosin heavy chain (MyHC) increased during this process (Figure 1a), suggesting that C2C12 cells had successfully differentiated. Notably, the expression of cleaved caspase‐3 (CASP3) (Figure 1a) and the proportion of apoptotic C2C12 cells increased with differentiation (Figure 1b). Next, ZVAD, a pan‐caspase inhibitor, was used to inhibit apoptotic activity. Since ZVAD was reported to affect the occurrence of autophagy, which is of great significance for myogenic differentiation [17, 30], we evaluated the effect of ZVAD on autophagy during skeletal muscle differentiation. The results showed that ZVAD had no effect on the LC3B‐II/LC3‐I ratio and p62 expression (*p* > 0.05) during myogenic differentiation (Figure S1a). Meanwhile, MyHC was significantly decreased (*p* = 0.0029) after ZVAD treatment (Figure 1c). To rule out the nonspecific effects of ZVAD, CASP3 expression was knocked down by siRNA transfection. Consistent with the results of ZVAD treatment, knockdown of CASP3 also significantly inhibited (*p* < 0.01) the differentiation of C2C12 cells (Figure S1b). These results are in accordance with previous studies [26, 27], indicating that the elevated apoptotic activity is critical for myogenic differentiation and myotube formation.

To explore the mechanism by which apoptotic activity regulates C2C12 differentiation, proteomics sequencing was performed on C2C12 cells after they were treated with ZVAD. We identified 8539 and 8491 proteins in the Con and ZVAD groups, respectively (Figure S2a and Table S3). PCA analysis showed that ZVAD significantly altered the proteomic profile of C2C12 cells (Figure S2b). Differential expression analysis identified 126 differentially expressed proteins (DEPs), among which 90 were downregulated and 36 were upregulated (Figure S2c and Table S4). LC3B and P62 were not mapped in DEPs (Table S4), which further confirms that the effect of ZVAD on cellular differentiation is independent of the occurrence of autophagy. Gene set enrichment analysis (GSEA) showed that ZVAD significantly enriched the ‘DNA replication’ and ‘G1 to S cell cycle control’ pathways (Figure 1d). When myogenic differentiation was initiated, myoblasts exited the cell cycle and entered a quiescent state [31]. This exit from the cell cycle is a crucial step as it allows the cells to withdraw from proliferation and commit to the differentiation program. By detecting the changes in the cell cycle during the differentiation process, we found that the arrest in the G0/G1 phase occurred as early as 6 h after the induction of differentiation, while the decrease in the percentage of cells in the S phase took place 12 h after differentiation (Figure S2d). We then detected the changes in the cell cycle at 12 h after ZVAD treatment. The results showed that ZVAD delayed cell cycle exit and increased the percentage of C2C12 myoblasts in the S phase (Figure 1e).

KEGG analysis showed that the cytoskeleton in muscle cells, an essential pathway for maintaining the structural properties of muscle cells and regulating skeletal muscle cell differentiation, was suppressed after ZVAD treatment (Figure S2e and Table S5). Cellular component (CC) enrichment in GO analysis (Table S6) revealed that DEPs enriched in myofibril and sarcomere (green font), confirming that ZVAD treatment affects skeletal muscle development; unexpectedly, extracellular region, extracellular space and extracellular matrix (red font) were significantly mapped in CC enrichment (Figure 1f and Table S6). These results indicate that apoptotic activity regulates skeletal muscle cell differentiation by influencing multiple biological processes, such as cell cycle regulation and cytoskeletal dynamics. Of note, the extracellular region has piqued our interest.

### 3.2 Isolation and Characterisation of C2C12‐Derived apoVs

**FIGURE 2:**
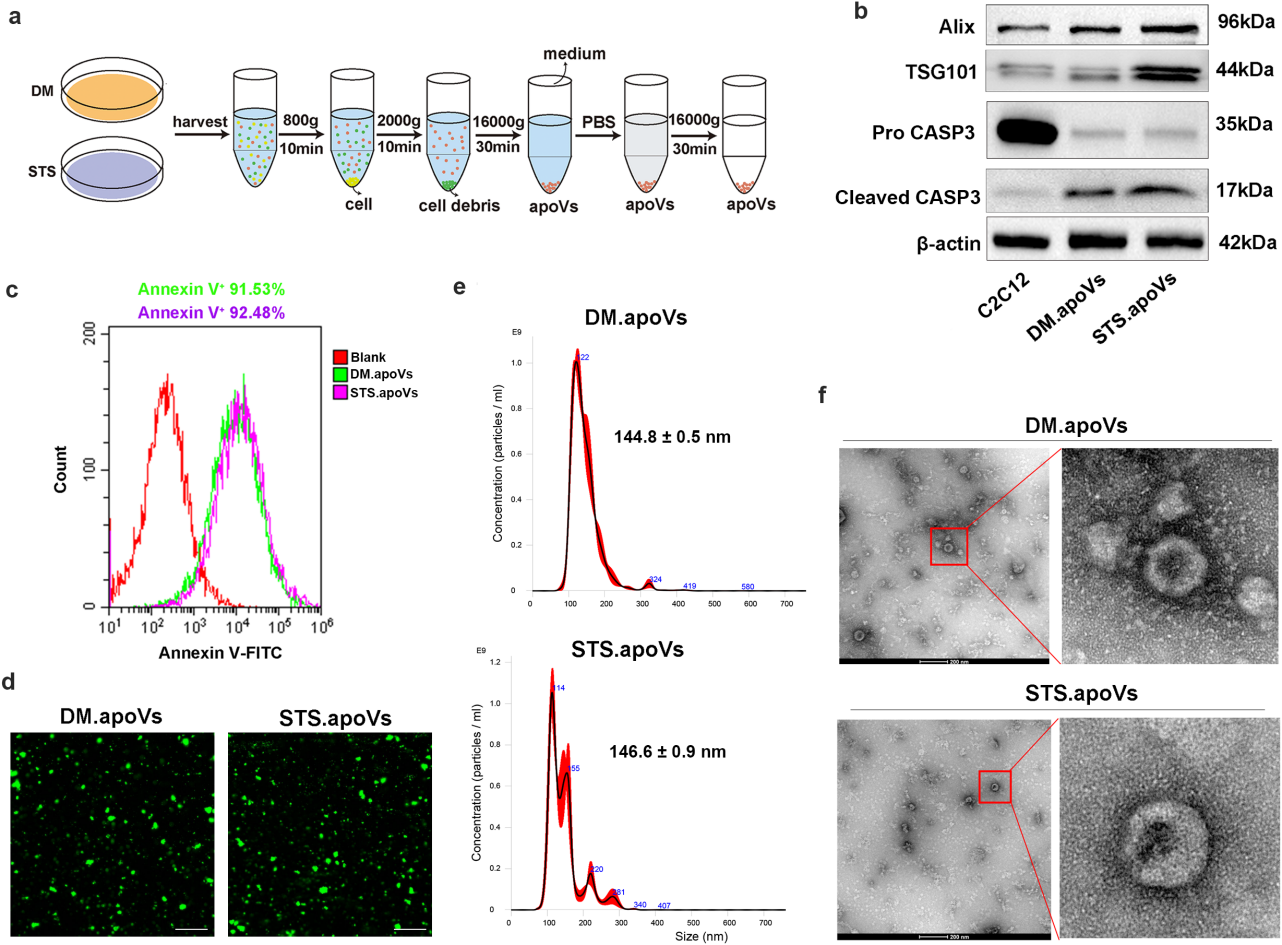
Isolation and characterisation of C2C12‐derived apoVs. (a) Schematic drawing of the gradient centrifugation protocol for isolating apoptotic extracellular vesicles (apoVs) from differentiated C2C12 cells (DM.apoVs) or STS‐treated undifferentiated C2C12 myoblasts (STS.apoVs). (b) The protein expression of CASP3, Alix, TSG101 in C2C12 myoblasts, DM.apoVs and STS.apoVs was determined by western blot analysis, and β‐actin was used as the loading control. (c) Flow cytometric analysis of Annexin V‐FITC staining in DM.apoVs and STS.apoVs. (d) Representative confocal microscopy images of Annexin V‐FITC staining in DM.apoVs and STS.apoVs. Scale bars, 10 μm. (e) The size distribution of DM.apoVs and STS.apoVs was determined by nanoparticle tracking analysis (NTA). (f) Representative transmission electron microscope (TEM) images showing the morphology of apoVs. Scale bar, 200 nm.

We next explored extracellular components closely related to cellular apoptosis. Studies have found that apoptotic cells can generate numerous apoVs [29], and we therefore hypothesised that apoVs may be produced during the process of myogenic differentiation. To verify this conjecture, we isolated DM.apoVs using a sequential differential centrifugation method, with STS‐induced apoVs serving as the positive control (Figure 2a). Western blot analysis showed that both DM.apoVs and STS.apoVs highly expressed the general apoV markers cleaved CASP3, Alix and TSG101 (Figure 2b). Moreover, these proteins were undetectable in the non‐vesicular control group, further confirming that they were associated with vesicles rather than free proteins (Figure S3a). Flow cytometry and immunofluorescence staining revealed the exposure of PtdSer on DM.apoVs and STS.apoVs (Figure 2c–d). NTA results showed that the median size of DM.apoVs was 144.8 ± 0.5 nm, similar to that of STS.apoVs (146.6 ± 0.9 nm) (Figure 2e), and both had a particle‐to‐protein ratio greater than 10^9^ particles/μg (Figure S3b), suggesting that apoVs possess excellent purity and high enrichment quality. Both DM.apoVs and STS.apoVs showed a typical double‐membrane spherical structure as shown by TEM (Figure 2f). These results indicate that apoVs are produced during the differentiation process of skeletal muscle cells.

### 3.3 Caspase Inhibition Hinders Myogenic Differentiation by Reducing apoV Production

**FIGURE 3:**
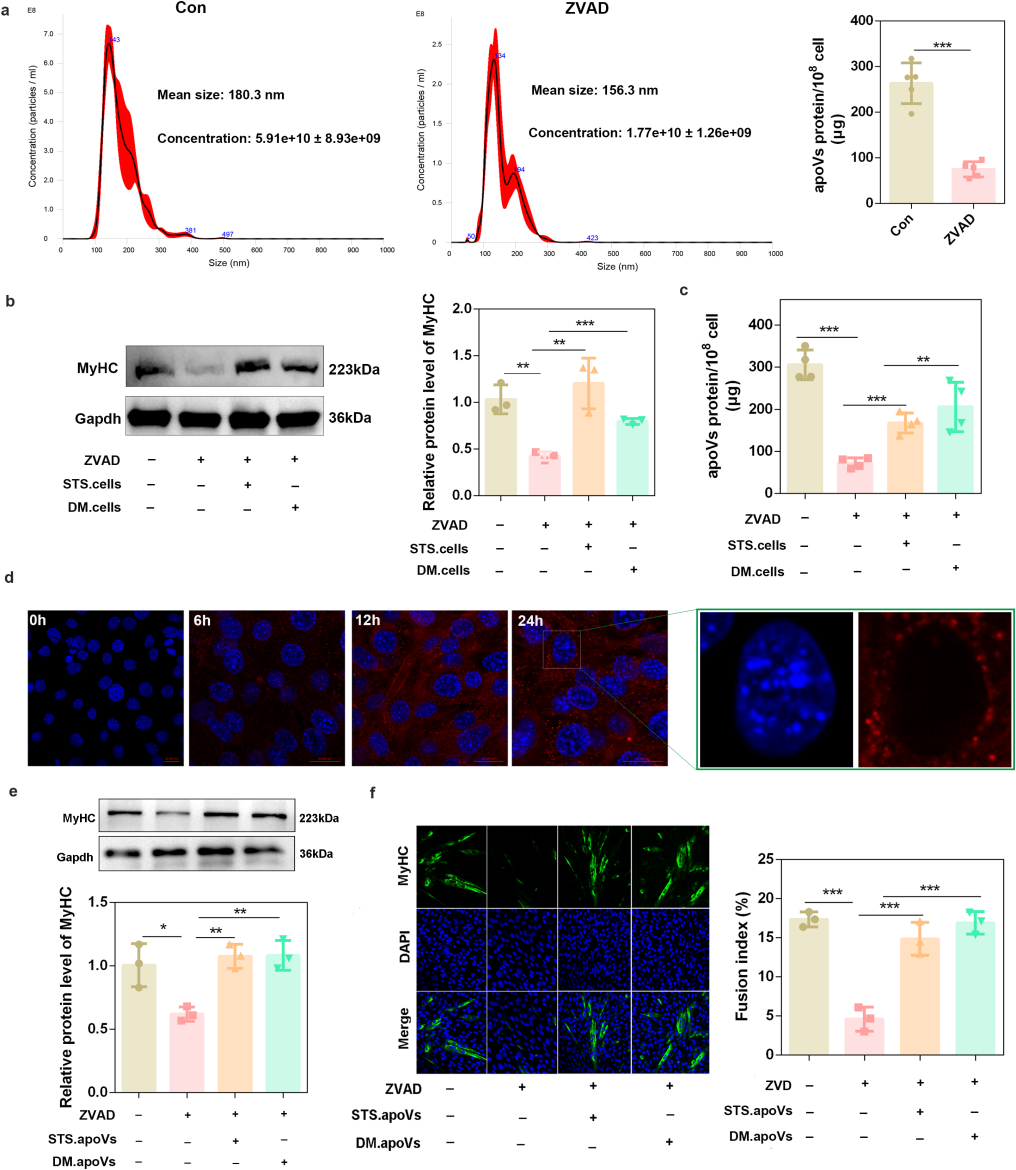
Caspase inhibition hinders myogenic differentiation by reducing apoVs production. (a) C2C12 myoblasts pre‐treated with 50 μM ZVAD were induced to differentiate for 1 days, and then, the apoVs were isolated from differentiation medium. Size distribution and the concentration of apoVs were determined by NTA; the total apoVs protein level was determined by BCA analysis in ZVAD pre‐treated C2C12 cells (*n* = 3). (b) Staurosporine (STS)‐derived apoptotic cells (STS.cells) or DM‐derived apoptotic cells (DM.cells) were collected and used to treat ZVAD pre‐treated C2C12 myoblasts. The protein level of MyHC was detected by western blot analysis after 3 days of differentiation. Gapdh was used as the loading control, and protein signal intensities were analysed using the ImageJ software (*n* = 3). (c) The total apoVs protein level was determined by BCA analysis after the indicated treatments (*n* = 3). (d) Representative confocal microscopy images showing the uptake of PKH26‐labelled apoVs (red) in C2C12 myoblasts, counterstained by DAPI (blue). Scale bars, 20 μm. (e) STS.apoVs or DM.apoVs were used to treat ZVAD pre‐treated C2C12 myoblasts. The protein level of MyHC was detected by western blot analysis after 3 days of differentiation. Gapdh was used as the loading control, and protein signal intensities were analysed using the ImageJ software (*n* = 3). (f) STS.apoVs or DM.apoVs were used to treat ZVAD pre‐treated C2C12 myoblasts, and the fusion index of C2C12 cells was determined by MyHC immunofluorescence staining. MyHC, green. Nuclei, blue. Scale bars, 50 μm. *p < 0.05; **p < 0.01; ***p < 0.001.

As shown in Figure 1, caspase inhibition affected C2C12 differentiation and extracellular space. Since apoVs belonged to extracellular components, we next explored whether apoptotic activity affects C2C12 differentiation by generating apoVs. The results showed that ZVAD decreased the concentration of apoVs and their protein levels (*p* < 0.0001) (Figure 3a). ApoVs are released by apoptotic cells, and we found that adding STS‐derived apoptotic cells (STS.cells) or DM‐derived apoptotic cells (DM.cells) to C2C12 cells rescued the impaired differentiation (Figure 3b) and increased apoV protein levels (Figure 3c) compared with the ZVAD group. These results suggest that ZVAD may hinder myogenic differentiation by reducing apoV production, whereas apoptotic cells rescue differentiation by replenishing apoVs.

Using PKH26‐labelled apoVs, we confirmed that C2C12 cells internalise apoVs, and apoVs would accumulate around the nucleus (Figure 3d). Western blot and immunofluorescence analyses showed that C2C12‐derived apoVs ameliorated the myogenic differentiation defects (*p* < 0.01) induced by caspase inhibition (Figure 3e–f and Figure S4). These results demonstrate that the production of apoVs is dependent on caspase activity, and apoptotic activity modulates myogenic differentiation by generating apoVs.

### 3.4 C2C12‐Derived apoVs Contain Multiple pro‐Differentiation Pathways

**FIGURE 4:**
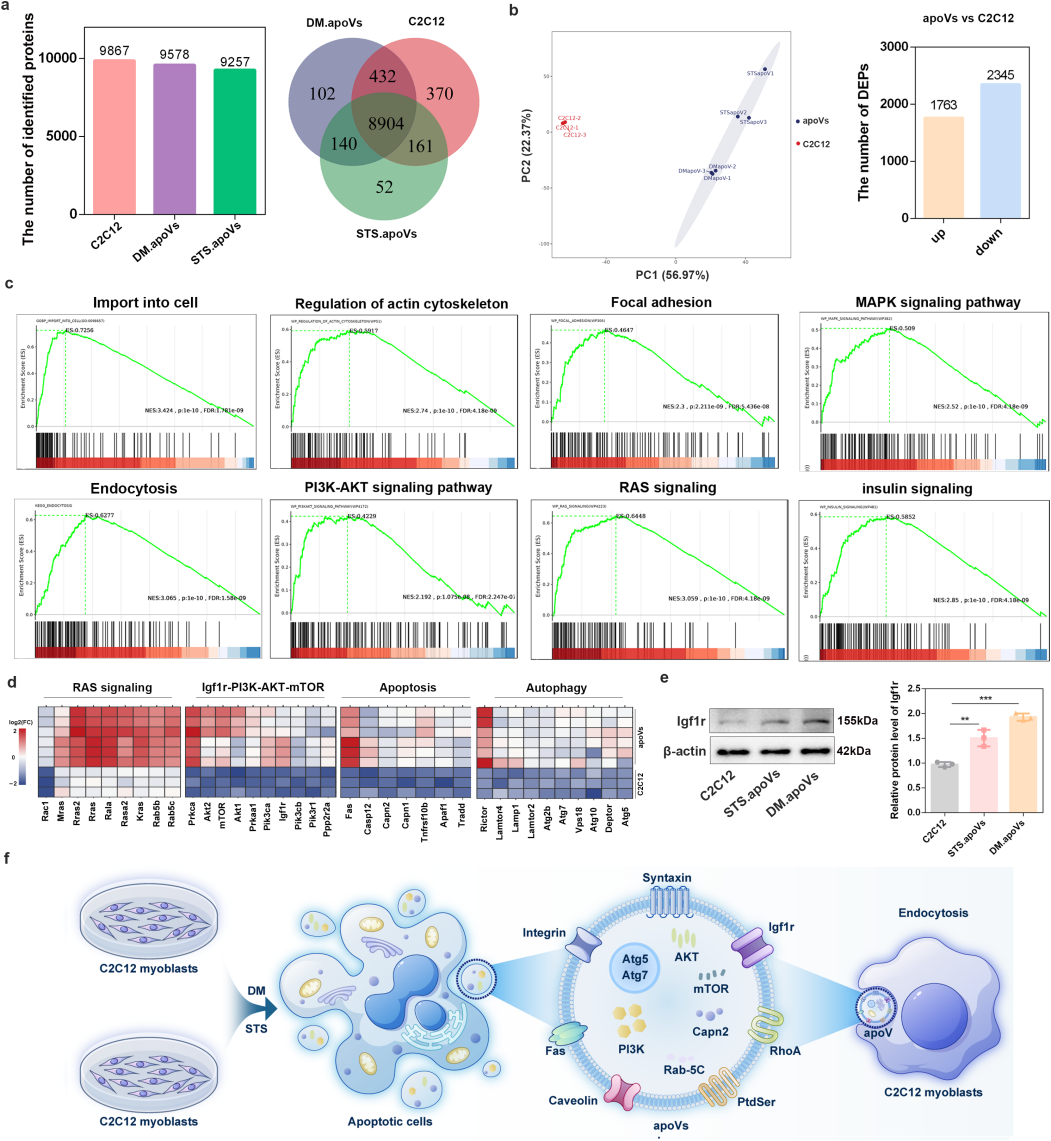
C2C12‐derived apoVs contain multiple pro‐differentiation pathways. (a) The number of identified proteins in the C2C12 myoblasts, DM.apoVs and STS.apoVs group and Venn diagram analysis between three groups. (b) Principal component analysis (PCA) and the number of DEPs of C2C12 myoblasts and C2C12‐derived apoVs. (c) GSEA was performed using the DEPs between the C2C12 group and the apoVs group. (d) Heatmap analysis of RAS signalling, Igf1r/PI3K/AKT/mTOR pathway, apoptosis and autophagy‐related genes in each group. (e) The protein expression of Igf1r was determined by western blot analysis, and β‐actin was used as the loading control. (f) Schematic drawing of the absorption and uptake of C2C12‐derived apoVs.

ApoVs contain proteins, DNA fragments, miRNAs, lipids and other components. Studies have shown that apoV‐associated proteins play critical roles in cellular communication and immune regulation [29]. To investigate how C2C12‐derived apoVs exert their functions, we performed proteomic analysis on parental C2C12 myoblasts, DM.apoVs and STS.apoVs. As shown in Figure 4a, we identified 9867, 9578, and 9257 proteins in parental C2C12 cells, DM.apoVs and STS.apoVs, with 8904 proteins shared among all three groups. This indicates that apoVs inherit most proteins from parental C2C12 cells. Hierarchical clustering analysis showed that DM.apoVs clustered with STS.apoVs, and both were distinct from C2C12 myoblasts (Figure S5a), suggesting that C2C12‐derived apoVs possess a unique proteomic profile compared with parental cells.

We therefore combined sequencing data from DM.apoVs and STS.apoVs into ‘apoVs’ (Figure 4b). Differential expression analysis identified 1763 upregulated and 2345 downregulated proteins in apoVs versus the parental C2C12 cells (Figure 4b and Table S7). By randomly selecting 20 highly expressed proteins in apoVs and comparing them with Vesiclepedia databases (Table S8), we found that all these proteins have been identified in different types of extracellular vesicles (EVs). This indicates that the protein components in apoVs are intrinsic components of the vesicles. GSEA revealed that ‘cell import’ and ‘endocytosis’ pathways were significantly enhanced in apoVs (Figure 4c), confirming the uptake of apoVs observed in Figure 3e. More importantly, we found that apoVs contained multiple pro‐differentiation pathways and related components, including the actin cytoskeleton, autophagy, PI3K‐AKT, MAPK, insulin and mTOR signalling pathways (Figure 4c,d). Additionally, apoptosis‐associated proteins, such as FAS, Tradd, Tnfrsf10b and Apaf1, were enriched in apoVs (Figure 4d).

While both DM.apoVs and STS.apoVs exhibit general characteristics of apoptotic vesicles and express pro‐differentiation signals, differential expression analysis identified 2534 DEPs between them (Figure S5b and Table S9), with enrichment in mitochondrial metabolism‐related proteins (Figure S5c and Table S10). DM.apoVs originate from differentiating C2C12 cells, whereas STS.apoVs derive from undifferentiated myoblasts. Skeletal muscle cell differentiation is accompanied by significant changes in mitochondrial energy metabolism [32], indicating that the proteomic profile of apoVs reflects the physiological state of their parental cells.

Igf signalling pathway is the core associated with skeletal muscle development [33]. Skeletal muscle myoblasts have the ability to secrete Igfs, which act on themselves or adjacent cells through autocrine/paracrine mechanisms and coordinately regulate myoblast proliferation, differentiation and myotube maturation by activating Igf1r‐related signalling pathways, such as PI3K/AKT/mTOR [33]. Our sequencing results indicate that apoVs can inherit a variety of Igf signalling pathway‐related proteins from their parental C2C12 cells, including Igf1r, Igfbp2 and Igfbp6 (Table S7). Of note, Igf1r levels in apoVs were significantly higher than those in their parental C2C12 cells (*p* < 0.01) (Figure 4e), which may explain the ability of apoVs to rescue differentiation defects (Figure 4f).

### 3.5 C2C12‐Derived apoVs Promoted Myogenic Differentiation Depending on Igf1r‐Mediated PI3K/AKT/mTOR Activation

**FIGURE 5:**
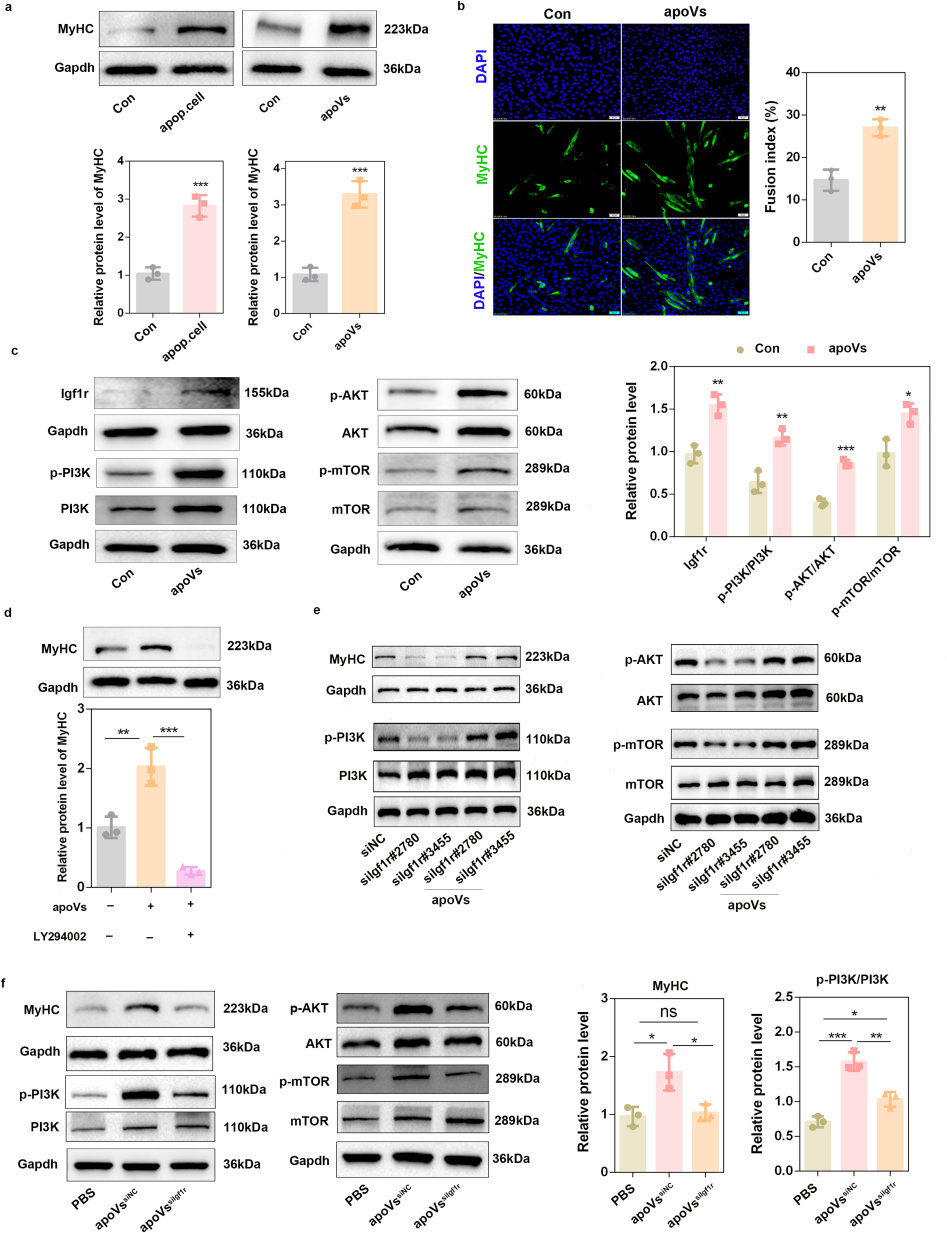
C2C12‐derived apoVs promoted myogenic differentiation depending on Igf1r‐mediated PI3K/AKT/mTOR activation. (a) Apoptotic cells generated from myogenic differentiation or apoVs isolated from differentiation medium were used to treat C2C12 myoblasts, and the MyHC expression was detected by western blot analysis after 3 days of differentiation. Gapdh was used as the loading control, and protein signal intensities were analysed using the ImageJ software (*n* = 3). (b) ApoVs isolated from differentiation medium were used to treat C2C12 myoblasts, and the fusion index of C2C12 cells was determined by MyHC immunofluorescence staining. MyHC, green. Nuclei, blue. Scale bars, 50 μm. (c) ApoVs isolated from differentiation medium were used to treat C2C12 myoblasts, and the protein level of Igf1r, p‐PI3K, PI3K, AKT, p‐AKT, mTOR and p‐mTOR was detected by western blot analysis after 3 days of differentiation. Gapdh was used as the loading control, and protein signal intensities were analysed using the ImageJ software (*n* = 3). (d) C2C12 myoblasts were co‐treated with 10 μM PI3K inhibitor (LY294002) and apoVs. After 3 days of differentiation, the protein level of MyHC was detected by western blot analysis. Gapdh was used as the loading control, and protein signal intensities were analysed using the ImageJ software (*n* = 3). (e) C2C12 cells were transfected with Igf1r siRNAs for 24 h and then induced to differentiation with normal or apoV‐containing DM for 3 days. The protein levels of MyHC, p‐PI3K, PI3K, AKT, p‐AKT, mTOR, p‐mTOR were detected by western blot analysis after 3 days of differentiation. Gapdh was used as the loading control. Protein signal intensities were analysed using the ImageJ software (*n* = 3). (f) ApoVs^siNC^ or apoVs^siIgf1r^ were used to treat C2C12 myoblasts, and the protein level of MyHC, p‐PI3K, PI3K, AKT, p‐AKT, mTOR and p‐mTOR was detected by western blot analysis after 3 days of differentiation. Gapdh was used as the loading control, and protein signal intensities were analysed using the ImageJ software (*n* = 3). *p < 0.05; **p < 0.01; ***p < 0.001.

To further verify the role of apoVs and explore the related mechanisms, we detected MyHC expression after adding DM.apoVs to normal C2C12 cells, with apoptotic cells collected from the differentiation process as the positive control. The results showed that adding DM.apoVs to the DM significantly increased MyHC protein expression (*p* = 0.0005) (Figure 5a), which was similar to the effect of apoptotic myoblasts (*p* = 0.0007). In addition, immunofluorescence results showed that apoVs significantly promoted the fusion index of C2C12 myoblasts (*p* < 0.01) (Figure 5b). Western blot analysis showed that apoVs elevated Igf1r protein levels and promoted PI3K/AKT/mTOR activation in both normal and ZVAD‐treated C2C12 cells (Figure 5c and Figure S6a); co‐treatment of apoVs with LY294002, the PI3K inhibitor, reduced MyHC expression (Figure 5d), suggesting that apoVs promote the differentiation of skeletal muscle cells through the activation of PI3K and its downstream signalling.

To explore the mechanism by which apoVs activate the PI3K signalling pathway in recipient cells, we transfected C2C12 cells with Igf1r siRNAs (Figure S6b) and then treated these Igf1r‐knockdown cells with apoVs. The results demonstrated that Igf1r knockdown significantly reduced MyHC expression and PI3K signalling activation relative to the control group (Figure 5e and Figure S6c); apoVs reversed the impairment of the PI3K pathway induced by Igf1r knockdown, with no statistically significant differences in these parameters compared with the control group (Figure 5e and Figure S6c). Then, we collected apoVs from Igf1r‐knockdown C2C12 cells and named them apoVs^siIgf1r^ (Figure S6d). The results showed that compared with apoVs^siNC^, the treatment of C2C12 cells with apoVs^siIgf1r^ reduced PI3K/AKT/mTOR activation and MyHC expression (Figure 5f and Figure S6e). These results revealed that C2C12‐derived apoVs promote myogenic differentiation by carrying Igf1r and activating the PI3K/AKT/mTOR pathway. Although the pro‐differentiation effect of apoVs was completely reversed after Igf1r knockdown (Figure 5f), the activation level of the PI3K was still higher than that in the PBS‐treated group (Figure 5f), suggesting that besides Igf1r, apoVs could also promote PI3K activation through other cargo components.

### 3.6 C2C12‐Derived apoVs Promoted Skeletal Muscle Hypertrophy in Vivo

**FIGURE 6:**
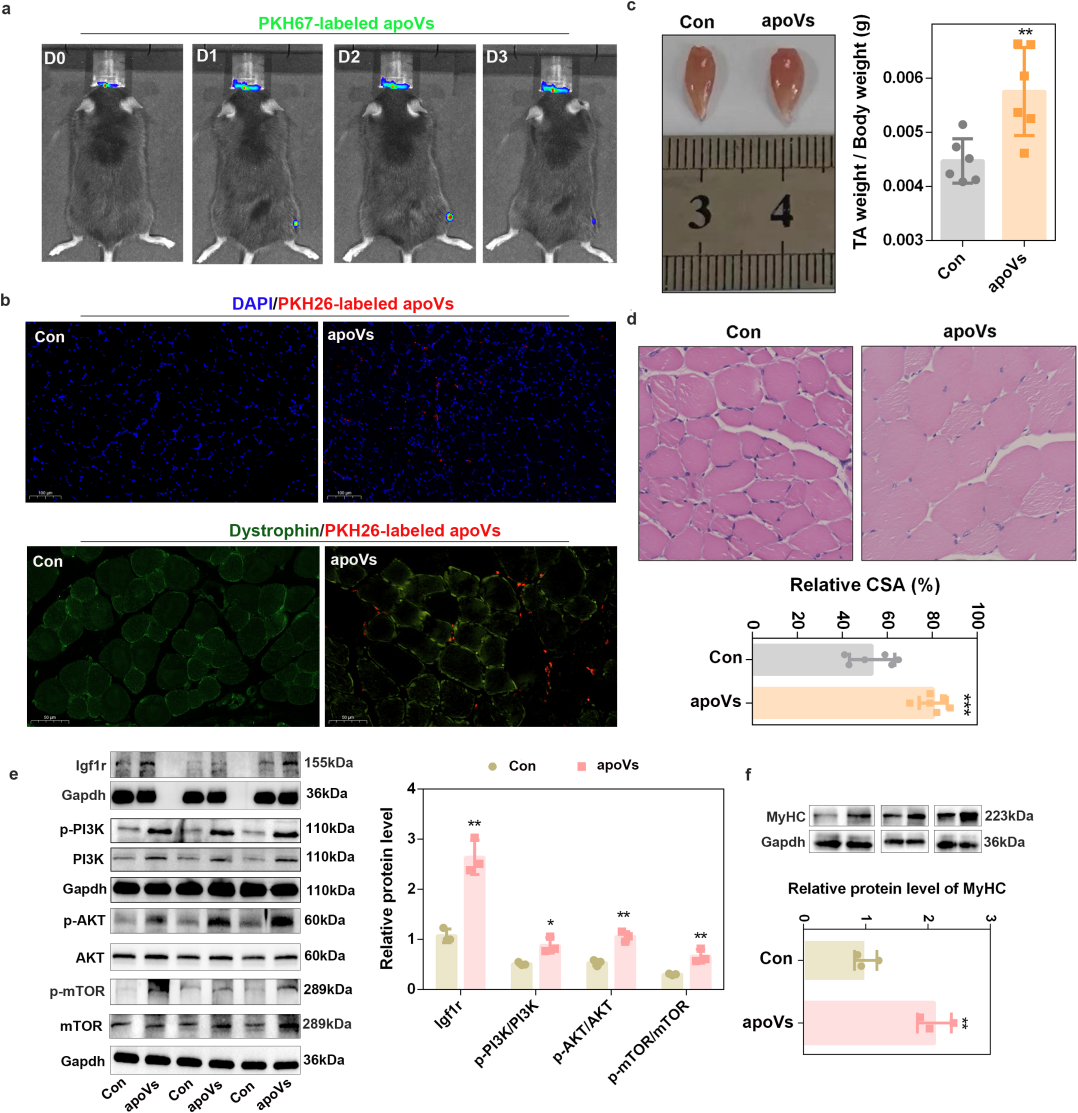
C2C12‐derived apoVs promoted skeletal muscle hypertrophy in vivo. (a) Representative whole‐body bioluminescence images of mouse after injecting PKH67‐labelled apoVs in tibialis anterior (TA) muscle. (b) Representative confocal microscopy images showing the uptake of apoVs. Red, PHK26‐labelled apoVs. Blue: nuclei. Green: dystrophin. Scale bar, 100 μm. (c) Representative images of TA muscles isolated from apoV‐injected 8‐week‐old mice; the ratio of TA muscle weight to body weight was calculated (*n* = 6). (d) H&E staining images of TA muscles isolated from apoVs‐injected 8‐week‐old mice, and the relative area was assessed using the ImageJ software (*n* = 6). (e) The protein level of Igf1r, p‐PI3K, PI3K, AKT, p‐AKT, p‐mTOR and mTOR was detected by western blot analysis in apoV‐injected 8‐week‐old mice. Gapdh was used as the loading control, and protein signal intensities were analysed using the ImageJ software (*n* = 3). (f) The protein level of MyHC was detected by western blot analysis in apoV‐injected 8‐week‐old mice. Gapdh was used as the loading control, and protein signal intensities were analysed using the ImageJ software (*n* = 3). *p < 0.05; **p < 0.01; ***p < 0.001.

Next, we injected DM.apoVs into the TA muscle of 8‐week‐old male mice. The results of in vivo imaging showed that the fluorescence intensity of apoVs reached the maximum at 2 days after injection (Figure 6a). By performing immunofluorescence analysis after isolating the TA muscle, we found significant accumulation of apoVs in the TA muscle; co‐staining of dystrophin and apoVs by confocal microscopy showed that apoVs were taken up by myofibres at 2 days post‐injection (Figure 6b). Next, DM.apoVs were injected into the TA muscle continuously for 8 weeks at 2 μg/g, and our results showed that apoVs caused a marked increase in the TA weight and the ratio of TA muscle to body weight (Cohen's d = 1.993, power = 0.874) (Figure S7a and Figure 6c). H&E staining results showed that apoVs significantly increased the relative cross‐sectional area (CSA) of the skeletal muscle (*p* = 0.0002) (Figure 6d). Meanwhile, apoV injection upregulated Igf1r protein level and enhanced the activation of PI3K/AKT/mTOR (Figure 6e); the protein expression of MyHC was also significantly increased (Figure 6f) in apoV‐treated mice (*p* = 0.0043). All these results illustrated that C2C12‐derived apoVs significantly promote skeletal muscle hypertrophy.

### 3.7 C2C12‐Derived apoVs Attenuated age‐Related Decline in Muscle Mass and Function

**FIGURE 7:**
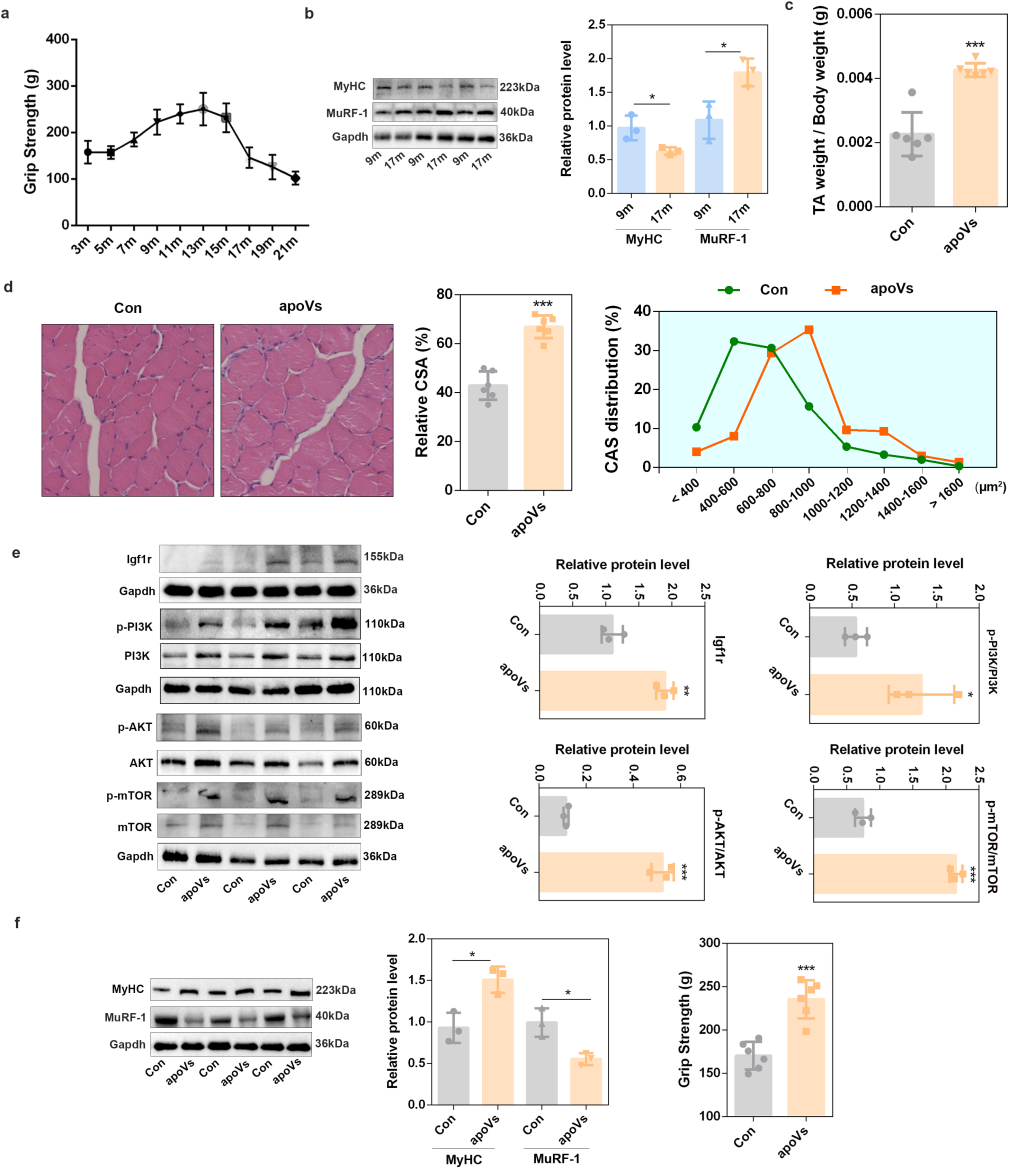
C2C12‐derived apoVs alleviated age‐related muscle loss. (a) The grip strength of mice at different months of age. (b) The protein level of MyHC and MuRF‐1 was detected by western blot analysis in 9‐month‐old and 17‐month‐old mice (*n* = 3). (c) The ratio of TA muscle weight to body weight was assessed in apoVs‐injected aged mice (*n* = 6). (d) H&E staining images of TA muscles isolated from apoV‐injected aged mice, and the relative area was assessed using the ImageJ software (*n* = 6). (e) The protein level of Igf1r, p‐PI3K, PI3K, AKT, p‐AKT, p‐mTOR and mTOR was detected by western blot analysis in apoV‐injected aged mice. Gapdh was used as the loading control, and protein signal intensities were analysed using the ImageJ software (*n* = 3). (f) The protein level of MyHC and MuRF‐1 was detected by western blot analysis in apoV‐injected aged mice. Gapdh was used as the loading control, and protein signal intensities were analysed using the ImageJ software (*n* = 3). The grip strength of apoV‐injected aged mice (*n* = 6). *p < 0.05; **p < 0.01; ***p < 0.001.

The mTOR signalling pathway regulates the synthesis of muscle protein, and its hypo‐activation results in decreased muscle protein synthesis and increased protein degradation, ultimately resulting in the decline of muscle mass and strength [32]. C2C12‐derived apoVs highly expressed mTOR, indicating that they may play a role in alleviating muscle loss. By detecting muscle strength during the aging process, we found that the 17‐month‐old mice exhibited a significant decrease in muscle strength (Figure 7a); compared with 9‐month‐old mice, the aging‐related proteins, such as p21 and p53, in 17‐month‐old mice were significantly increased (Figure S7b); moreover, the relative CSA (Figure S7c) was also significantly reduced in 17‐month‐old mice. In addition, the protein levels of MyHC were decreased and the protein levels of MuRF‐1 were increased (Figure 7b), which indicates that muscle function begins to decline with increasing age. Next, DM.apoVs or PBS were injected into 15‐month‐old mice. After 8 weeks of injection, the TA muscles were isolated at 17 months old for detection. The results showed that apoVs caused a marked increase in the TA muscle weight and the ratio of TA muscle to body weight in 17‐month‐old mice (Cohen's d = 3.97, power = 0.99) (Figure S7d and Figure 7c). H&E staining results showed that apoVs significantly increased the relative CSA (*p* < 0.0001) and CSA distribution of the skeletal muscle in apoV‐treated aging mice (Figure 7d). Meanwhile, DM.apoV injection significantly increased the activation of Igf1r/PI3K/AKT/mTOR signalling pathway (Figure 7e) and the protein expression of MyHC (*p* = 0.0141) (Figure 7f). In addition, DM.apoV injection decreased the protein levels of MuRF‐1 and increased grip strength in aged mice (Figure 7f). All these results suggest that C2C12‐derived apoVs alleviate age‐related decline in muscle mass and function.

## 4 Discussion

More and more evidence indicates that apoptosis is an integral and highly coordinated mechanism that significantly impacts the proper progression of skeletal muscle cell differentiation [18, 20, 26]. Despite the well‐defined role of apoptotic activity or apoptosis‐related genes in promoting skeletal muscle cell differentiation, the mechanism by which apoptosis affects myogenic differentiation is unclear. In this study, we found that ZVAD significantly inhibits the differentiation of C2C12 cells. Then, we further knocked down CASP3, and similarly observed a reduction in C2C12 differentiation. As a pan‐caspase inhibitor, ZVAD has been widely used in current research; however, researchers have found that ZVAD could regulate the occurrence of cellular autophagy [30], leading to non‐apoptosis‐related biological changes. In this study, the results revealed that ZVAD does not affect autophagy during the differentiation of C2C12 cells, which suggests that ZVAD may exert different effects in different cell types. Meanwhile, CASP3 knockdown further confirmed the crucial role of apoptotic activity in myogenic differentiation.

Treating C2C12 with ZVAD impeded skeletal muscle cells from exiting the cell cycle and affected actin cytoskeleton in muscle cells. The cell cycle controlling and actin cytoskeleton dynamics are two important events during myofibril differentiation. Some factors that regulate the cell cycle, such as microRNA‐206, can also be involved in myogenic differentiation [14]. In addition, cellular actin cytoskeleton dynamics regulate the activity of myocardin‐related transcription factors (MRTFs) and serum response factor (SRF) to regulate myogenesis [34]. What is most intriguing is that we found significant changes in extracellular components in GO enrichment, such as extracellular matrix, extracellular space and extracellular region, after inhibiting cell apoptotic activity. In recent years, the application of extracellular matrix on constructing artificial skeletal muscle tissue has been widely reported, and Liang et al. found that perfusable adipose decellularised extracellular matrix biological scaffold co‐recellularised with adipose‐derived stem cells and L6 promotes functional skeletal muscle regeneration following volumetric muscle loss [35]. These results explained the potential mechanisms by which apoptosis affects skeletal muscle cell differentiation from multiple perspectives, and provided new theoretical basis for the development and formation of skeletal muscle.

EVs are small lipid membrane vesicles that are secreted from cells into the extracellular space. EVs mainly include exosomes, microvesicles and apoVs [36]; of note, apoVs are bilayer lipid structures with unique biological and functional characteristics generated during apoptosis. They play a significant role in maintaining tissue and organ homeostasis [29]. ApoVs can inherit various CCs from their parental cells, including proteins, nucleic acids and lipids, and exert biological effects by carrying and delivering these components. ApoVs derived from fibroblast‐converted hepatocyte‐like cells effectively ameliorate liver fibrosis [37], and mesenchymal stem cells (MSC)–derived apoVs have a crucial effect on repairing DNA damage and suppress premature cellular senescence [38]. However, whether the increased apoptotic activity during myogenic differentiation would lead to the generation of apoVs, and whether apoVs could play a role in promoting the differentiation of skeletal muscle cells remains unclear. In this study, apoVs were extracted and identified from C2C12 cells (DM.apoVs or STS.apoVs). Although the periods, which DM.apoVs and STS.apoVs were derived from were different, they both expressed the general apoVs markers cleaved caspase 3, Alix and TSG101, and had similar protein expression profiles. Fas is a key functional protein exerting apoV effects [39], and our results showed that Fas is significantly highly expressed in C2C12‐derived apoVs, demonstrating that high expression of Fas, an apoptotic marker, is a key characteristic of apoVs.

In the comparison between ESCs and ESC‐derived apoVs, only a few hundred proteins have been identified as having differences [40]. This is consistent with our experimental results (9867 in parental C2C12 cells vs. 9578 in DM.apoVs vs. 9257 in STS.apoVs). In addition, we randomly selected multiple proteins detected in apoVs for comparison with Vesiclepedia databases and found that all these proteins have been identified in various types of EVs, further indicating that the identified proteins are intrinsic to apoVs and that apoVs can inherit most of the proteins from their parental cells. In fact, the role of exosomes in the differentiation of skeletal muscle cells has been studied [41]; studies have shown that there are significant differences between apoVs and exosomes in terms of functional protein cargo and surface markers [39]. Currently, little is known about the non‐apoptotic specific marker molecules of apoVs. Therefore, identifying apoV‐specific non‐apoptotic markers that can verify their purity by comparing apoVs derived from different cell types and by analysing the differences between apoVs and other EVs (e.g., exosomes) is crucial for studying the unique functions of apoVs, and it will also become the focus of our next work.

The Igf1r/PI3K/AKT/mTOR signalling pathway acts as a central regulator in skeletal muscle development, integrating growth signals to balance muscle protein synthesis and degradation. It is known that a variety of drugs promote skeletal muscle development or delay muscle atrophy by targeting this signalling pathway [42]. We found that apoVs carry abundant Igf1r, and its protein level in apoVs is significantly higher than that in parental C2C12 cells. After being taken up by recipient cells, apoVs can promote PI3K/AKT/mTOR activation. Knocking down Igf1r in recipient cells does not affect the function of apoVs, indicating that apoVs exert their function independently of recipient cells' Igf1r; however, knocking down Igf1r in apoVs significantly reduces their pro‐differentiation effects and the activation of downstream signals. These results revealed that apoVs promote the activation of PI3K in recipient cells by carrying and delivering Igf1r. Interestingly, although apoVs^siIgf1r^ reduces the activation of PI3K, its level remains significantly higher than that of the control group. Meanwhile, proteomic sequencing results of apoVs show that apart from Igf1r, apoVs contain abundant insulin and insulin receptors, the key upstream molecules that also activate the PI3K signalling pathway. In contrast to the activation of PI3K, the expression of MyHC can be completely reversed by apoVs^siIgf1r^, suggesting that the cargo of apoVs can activate the PI3K pathway through multiple routes to exert their corresponding functions. Meanwhile, the addition of LY294002, a PI3K inhibitor, also blocked the positive effect of apoVs on skeletal muscle cell differentiation. Researchers found that low‐concentration LY294002 can directly inhibit mTOR activity [43]. Since mTOR is a central regulator both of the PI3K signalling pathway and myogenic differentiation, the reduced differentiation efficiency could, in part, be attributed to mTOR suppression rather than exclusive PI3K inhibition. Both in vivo and in vitro experiments have demonstrated that apoVs significantly upregulate Igf1r protein levels. This further confirms that the abundant Igf1r carried by apoVs can be transferred to recipient cells and exert effects in them. Actually, skeletal muscle cells have the ability to secrete Igf, and further investigation into the activation status and spatial localisation of Igf1r in apoVs is necessary in the following research.

Beyond its capacity to rescue the impaired skeletal muscle differentiation caused by caspase inhibition, C2C12‐derived apoVs also promoted the normal myogenic differentiation process and muscle hypertrophy and relieved the age‐related muscle loss through the Igf1r/PI3K/AKT/mTOR signalling pathway. With increasing age, skeletal muscle mass and function decrease significantly, eventually leading to muscle atrophy, which is a key factor impairing the health of the elderly [44]. By detecting the CSA of muscle fibres, grip strength and senescence protein markers, we found that mice began to exhibit muscle wasting at 17 months of age. Moreover, injection of apoVs significantly alleviated the loss of skeletal muscle mass and strength caused by aging. These findings are of great significance for the application of skeletal muscle‐derived apoVs in the treatment of muscle‐related diseases, and it also indicates the potential application value of apoVs in the treatment of muscle atrophy. Since the commonly used muscle atrophy models include obese mice and elderly mice aged over 23 months, it is necessary and of great significance to further investigate the therapeutic effect and mechanism of apoVs in muscle atrophy models to clarify their role in clinically relevant muscle atrophy.

## 5 Conclusion

**FIGURE 8:**
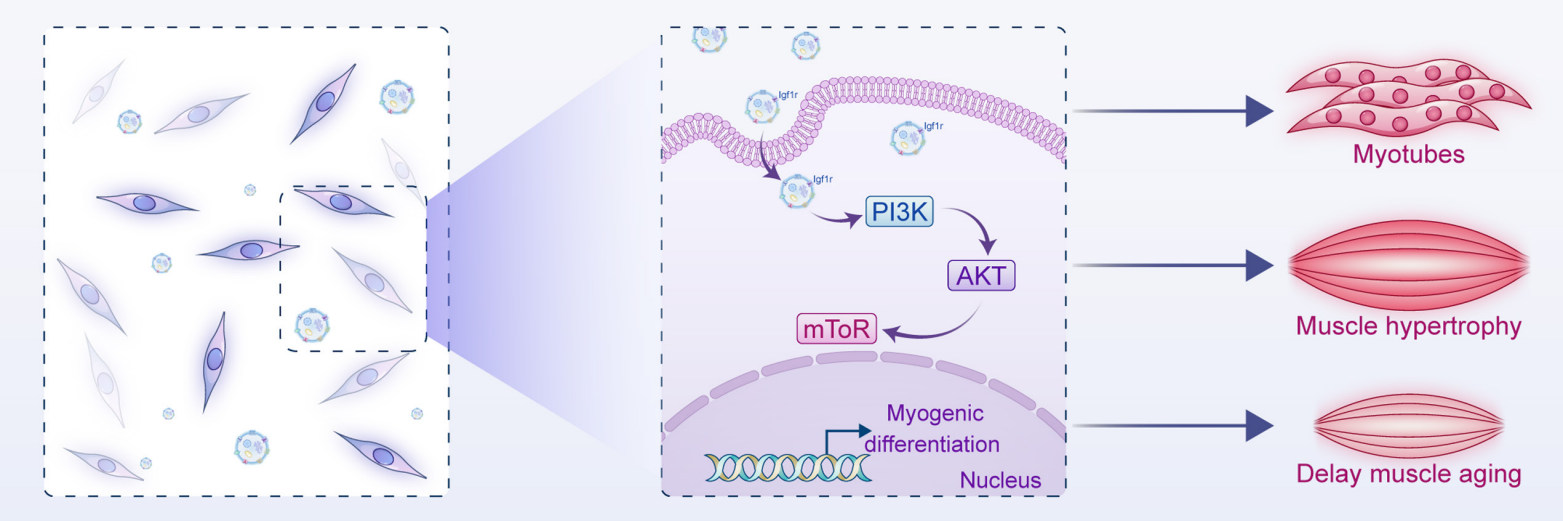
Schematic model of apoptotic activity modulates myogenic differentiation. During the differentiation of skeletal muscle cells, significant apoptosis occurs. Apoptotic cells can release apoptotic vesicles (apoVs), which carry abundant Igf1r. After being taken up by surrounding surviving cells, apoVs promote the activation of the PI3K/AKT/mTOR signalling pathway within the recipient cells, thereby enhancing myogenic differentiation, promoting skeletal muscle hypertrophy, and slowing age‐related muscle loss.

In conclusion, this study revealed that apoptotic activity is essential for skeletal muscle cells differentiation via generating apoVs (Figure 8). C2C12‐derived apoVs possess the fundamental characteristics of apoptotic vesicles and contain multiple pro‐differentiation signalling pathways. ZVAD inhibits the differentiation of skeletal muscle cells by suppressing the generation of apoVs, while the treatment of apoVs promotes skeletal muscle differentiation in both normal and ZVAD‐treated C2C12 cells through the Igf1r/PI3K/AKT/mTOR axis. In addition, apoVs can also promote skeletal muscle hypertrophy and delay aging‐related muscle loss. These results lay a theoretical foundation for elucidating the mechanisms of skeletal muscle development and treating skeletal muscle‐related diseases.

## Ethics Statement

All procedures for mice were performed in accordance with the guidelines issued by the Animal Research Institute Committee of Yangzhou University (SCXK‐2022‐0009).

## Conflicts of Interest

The authors declare no conflicts of interest.

## Supporting information


**Figure S1:** Caspase inhibition reduced myogenic differentiation. (a) The protein level of p62 and LC3B was detected by western blot analysis after ZVAD treatment. Gapdh was used as the loading control, and protein signal intensities were analysed using the ImageJ software (*n* = 3). (b) The protein level of CASP3 and MyHC was detected by western blot analysis after CASP3 siRNA transfection. Gapdh was used as the loading control, and protein signal intensities were analysed using the ImageJ software (*n* = 3). **p < 0.01; ***p < 0.001.
**Figure S2:** Cell cycle and cytoskeleton in muscle cells were affected by ZVAD treatment. (a) Identified protein numbers in the Con group and the ZVAD group. (b) Principal component analysis (PCA) for the Con group and the ZVAD group. (c) Volcano plot shows the differential expressed proteins (DEPs) between the Con group and the ZVAD group. (d) C2C12 myoblasts were induce to differentiate for 0, 6, 12, 24 and 48 h, and then, cell cycle analysis was performed. Data were analysed using the ModFit32 software (n = 3). (e) Chord Diagram of Kyoto Encyclopedia of Genes and Genomes (KEGG) enrichment analysis for the DEPs between the Con group and the ZVAD group.
**Figure S3:** Purity identification of apoVs. (a) The protein levels of the general apoV markers, including cleaved CASP3, Alix and TSG101, were detected in the non‐vesicular control group and DM.apoV group. (b) The particle‐to‐protein ratios of DM.apoVs and STS.apoVs were provided by calculating the particle number and protein content.
**Figure S4:** Addition of apoVs rescued the impaired myotube formation induced by caspase3 knockdown. (a) The protein level of MyHC was detected by western blot analysis after caspase 3 knockdown. Gapdh was used as the loading control. (b) Protein signal intensities were analysed using the ImageJ software (n = 3). ** p < 0.01.
**Figure S5:** Proteomic analysis reveals the difference between DM.apoVs and STS.apoVs. (a) Hierarchical clustering for C2C12 cells, DM.apoVs and STS.apoVs. (b) The number of DEPs between DM.apoVs and STS.apoVs. (c) GO analysis for the DEPs between DM.apoVs and STS.apoVs.
**Figure S6:** C2C12‐derived apoVs rescue the impaired myogenic differentiation induced by ZVAD via enhancing Igf1r/PI3K/AKT/mTOR pathway. (a) STS.apoVs or DM.apoVs were used to treat ZVAD pre‐treated C2C12 myoblasts. The protein level of Igf1r, PI3K, AKT, p‐AKT, mTOR and p‐mTOR was detected by western blot analysis after 3 days of differentiation. Gapdh was used as the loading control, and protein signal intensities were analysed using the ImageJ software (n = 3). (b) Igf1r knockdown efficiency in C2C12 cells was determined by western blot analysis. Gapdh was used as the loading control. Protein signal intensities were analysed using the ImageJ software (n = 3). (c) Data analysis for the MyHC expression and the ration of p‐PI3K/PI3K, p‐AKT/AKT and p‐mTOR/mTOR after C2C12 cells were co‐treated with siIgf1r and apoVs. (d) Igf1r knockdown efficiency in apoVs was determined by western blot analysis. β‐actin was used as the loading control for C2C12‐derived apoVs. Protein signal intensities were analysed using the ImageJ software (n = 3). (e) ZVAD pre‐treated C2C12 myoblasts were treated with apoVssiNCor apoVssiIgf1r. After 3 days of differentiation, the protein level of p‐PI3K, PI3K, AKT, p‐AKT, mTOR and p‐mTOR was detected by western blot analysis. Gapdh was used as the loading control, and protein signal intensities were analysed using the ImageJ software (n = 3). *p < 0.05; **p < 0.01; ***p < 0.001, ns, not significant.
**Figure S7:** ApoV injection increases the weight of skeletal muscle. (a) The body weight and TA muscle weight was assessed in apoV‐injected 8‐week‐old mice (n = 6). (b) The protein level of p21 and p53 was detected by western blot for 9‐month‐old and 17‐month‐old mice. Gapdh was used as the loading control, and protein signal intensities were analysed using the ImageJ software (n = 3). (c) H&E staining images of TA muscles isolated from 9‐month‐old and 17‐month‐old mice, and the relative area was assessed using the ImageJ software (n = 6). (d) The body weight and TA muscle weight was assessed in apoV‐injected aged mice (n = 6). *p < 0.05; **p < 0.01; ***p < 0.001.

**Table S1:** Sequences of siRNAs.

**Table S2:** Antibody information.

**Table S3:** Proteins information between the Con group and ZVAD group.

**Table S4:** Differentially expressed proteins between the Con group and the ZVAD group.

**Table S5:** Downregulated pathways of KEGG enrichment for DEPs between the ZVAD group and the Con group.

**Table S6:** GO enrichment of DEPs between the Con group and the ZVAD group.

**Table S7:** Differentially expressed proteins between the C2C12 group and the C2C12‐derived apoV group.

**Table S8:** Comparison of proteins in apoVs with Vesiclepedia database.

**Table S9:** Differentially expressed proteins between DM‐apoVs and STS‐apoVs.

**Table S10:** GO enrichment of DEPs between DM‐apoVs and STS‐apoVs.

## Data Availability

Proteomic sequencing data for ZVAD‐treated C2C12 cells have been submitted to iProX with PXD number: PXD064551. Proteomic sequencing data for C2C12‐derived apoVs have been submitted to iProX with PXD number: PXD064549.
