# Supplementary material for: C2C12‐Derived ApoVs Promote Skeletal Muscle Development and Ameliorate Age‐Related Muscle Loss Through Igf1r/PI3K/AKT/mTOR Pathway
Source: J Cachexia Sarcopenia Muscle. 2025 Dec 4;16(6):e70159. doi: 10.1002/jcsm.70159 (PMC12678004; doi:10.1002/jcsm.70159)
Supplement: Supplementary file 1 — Figure S1: Caspase inhibition reduced myogenic differentiation. (a) The protein level of p62 and LC3B was detected by western blot analysis after ZVAD treatment. Gapdh was used as the loading control, and protein signal intensities were analysed using the ImageJ software (n = 3). (b) The protein level of CASP3 and MyHC was detected by western blot analysis after CASP3 siRNA transfection. Gapdh was used as the loading control, and protein signal intensities were analysed using the ImageJ software (n = 3). **p < 0.01; ***p < 0.001. Figure S2: Cell cycle and cytoskeleton in muscle cells were affected by ZVAD treatment. (a) Identified protein numbers in the Con group and the ZVAD group. (b) Principal component analysis (PCA) for the Con group and the ZVAD group. (c) Volcano plot shows the differential expressed proteins (DEPs) between the Con group and the ZVAD group. (d) C2C12 myoblasts were induce to differentiate for 0, 6, 12, 24 and 48 h, and then, cell cycle analysis was performed. Data were analysed using the ModFit32 software (n = 3). (e) Chord Diagram of Kyoto Encyclopedia of Genes and Genomes (KEGG) enrichment analysis for the DEPs between the Con group and the ZVAD group. Figure S3: Purity identification of apoVs. (a) The protein levels of the general apoV markers, including cleaved CASP3, Alix and TSG101, were detected in the non‐vesicular control group and DM.apoV group. (b) The particle‐to‐protein ratios of DM.apoVs and STS.apoVs were provided by calculating the particle number and protein content. Figure S4: Addition of apoVs rescued the impaired myotube formation induced by caspase3 knockdown. (a) The protein level of MyHC was detected by western blot analysis after caspase 3 knockdown. Gapdh was used as the loading control. (b) Protein signal intensities were analysed using the ImageJ software (n = 3). ** p < 0.01. Figure S5: Proteomic analysis reveals the difference between DM.apoVs and STS.apoVs. (a) Hierarchical clustering for C2C12 cells, DM.apoVs an [file JCSM-16-e70159-s008.docx]

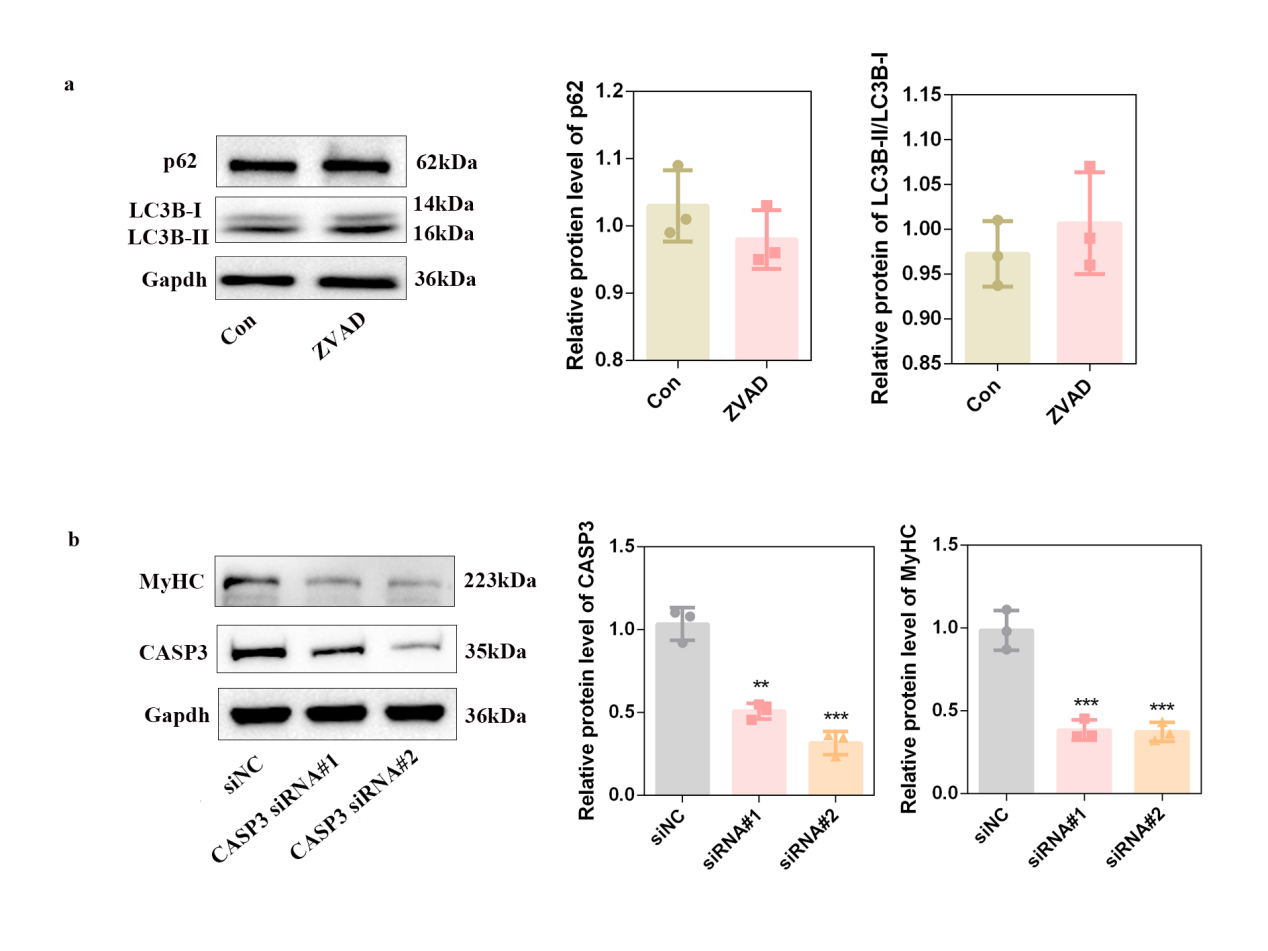


**Fig. S1. Caspase inhibition reduced myogenic differentiation.** **(a)** The protein level of p62 and LC3B was detected by western blot analysis after ZVAD treatment. Gapdh was used as the loading control, and protein signal intensities were analyzed using ImageJ software (n = 3). **(b)** The protein level of CASP3 and MyHC was detected by western blot analysis after CASP3 siRNAs transfection. Gapdh was used as the loading control, and protein signal intensities were analyzed using ImageJ software (n = 3). ** *P* < 0.01, ****P* < 0.001.


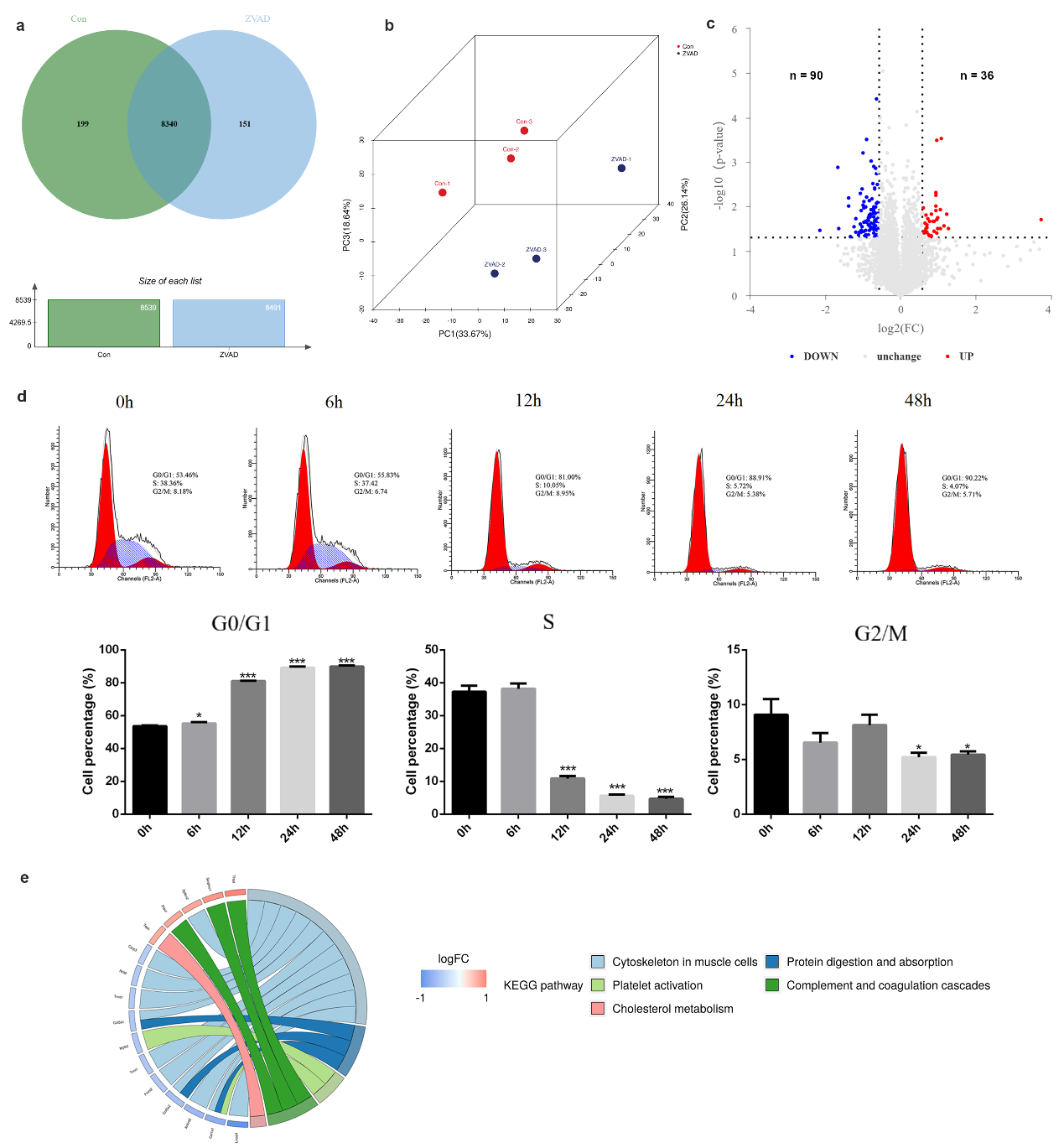


**Fig. S2. Cell cycle and cytoskeleton in muscle cells were affected by ZVAD treatment.** **(a)** Identified protein numbers in the Con group and the ZVAD group. **(b)** Principal component analysis (PCA) for the Con group and the ZVAD group. **(c)** Volcano plot shows the differential expressed proteins (DEPs) between the Con group and the ZVAD group. **(d)** C2C12 myoblasts were induce to differentiate for 0h, 6h, 12h, 24h and 48h, and then cell cycle analysis was performed. Data were analyzed using ModFit32 software (n = 3). **(e)** Chord Diagram of Kyoto Encyclopedia of Genes and Genomes (KEGG) enrichment analysis for the DEPs between the Con group and the ZVAD group.


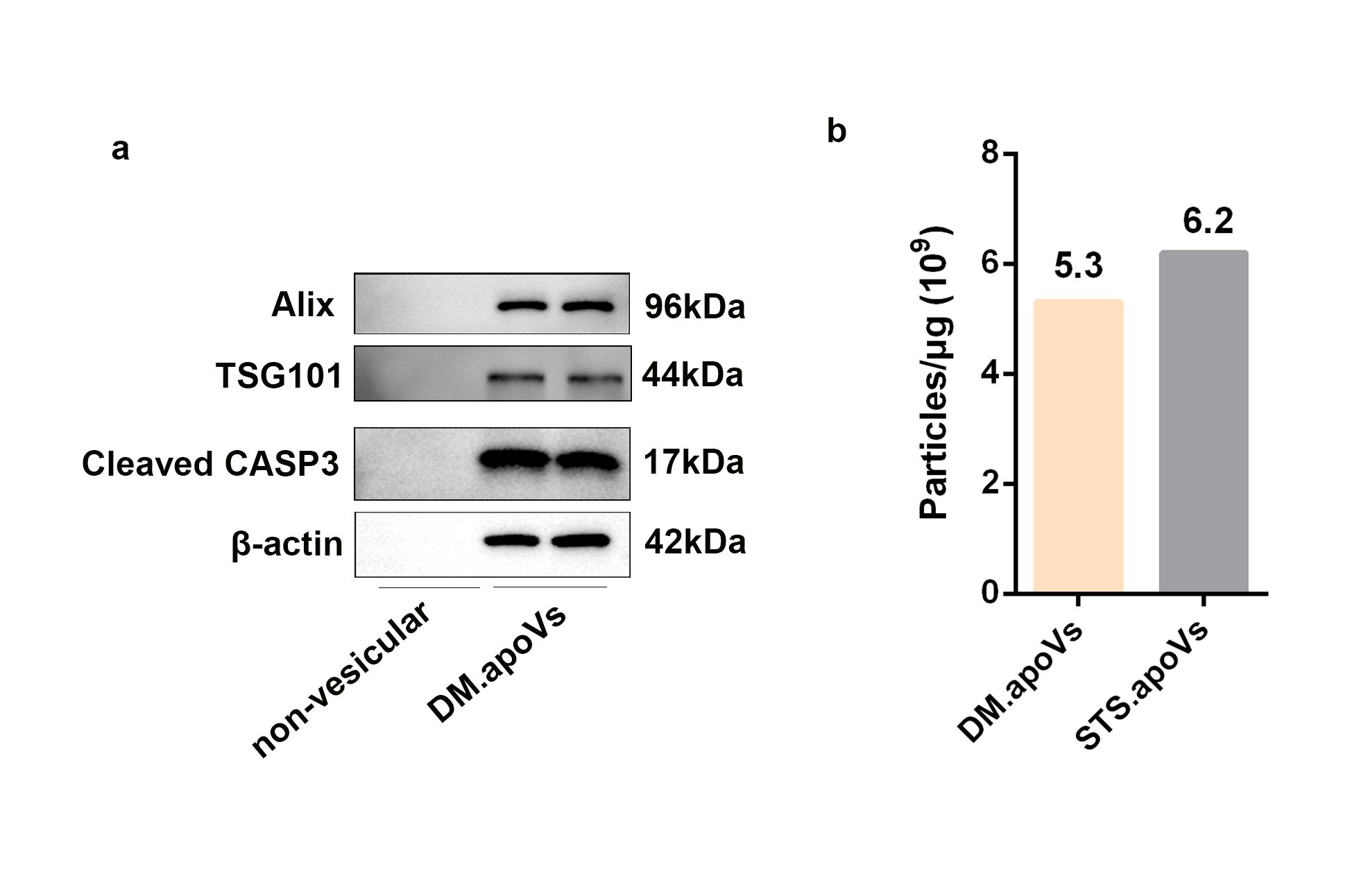


**Fig. S3. Purity identification of apoVs. (a)** The protein levels of the general apoVs markers, including cleaved CASP3, Alix and TSG101, were detected in the non-vesicular control group and DM.apoVs group. **(b)** The particle-to-protein ratios of DM.apoVs and STS.apoVs were provided by calculating the particle number and protein content.


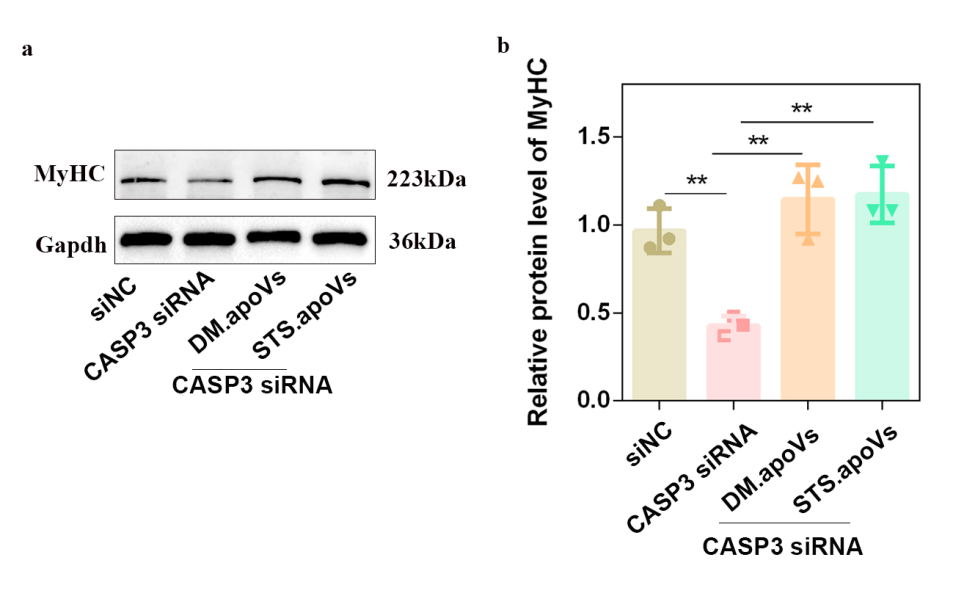


**Fig. S4. Addition of apoVs rescued the impaired myotube formation induced by caspase3 knockdown (a)** The protein level of MyHC was detected by western blot analysis after caspase 3 knockdown. Gapdh was used as the loading control. **(b)** Protein signal intensities were analyzed using ImageJ software (n = 3). ** *P* < 0.01.


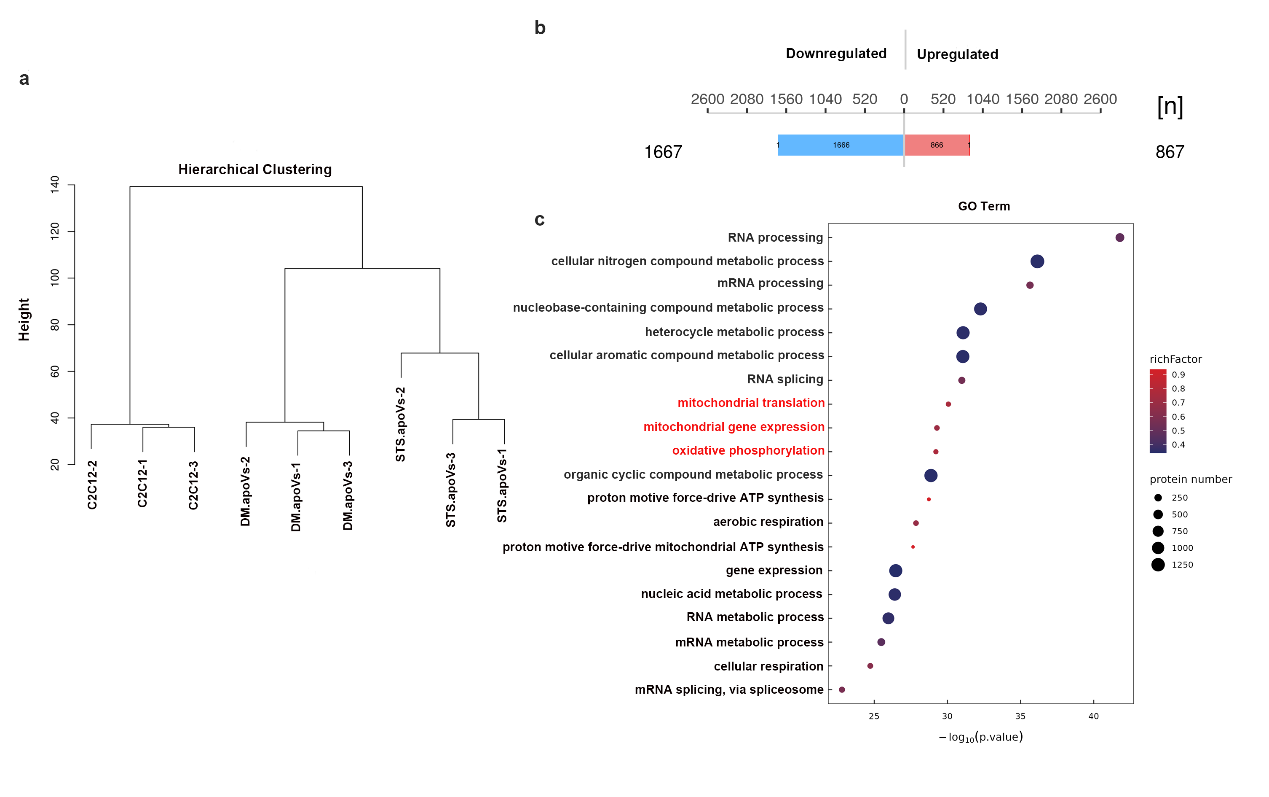


**Fig. S5. Proteomic analysis reveals the difference between DM.apoVs and STS.apoVs. (a)** Hierarchical clustering for C2C12 cells, DM.apoVs and STS.apoVs. **(b)** The number of DEPs between DM.apoVs and STS.apoVs. **(c)** GO analysis for the DEPs between DM.apoVs and STS.apoVs.


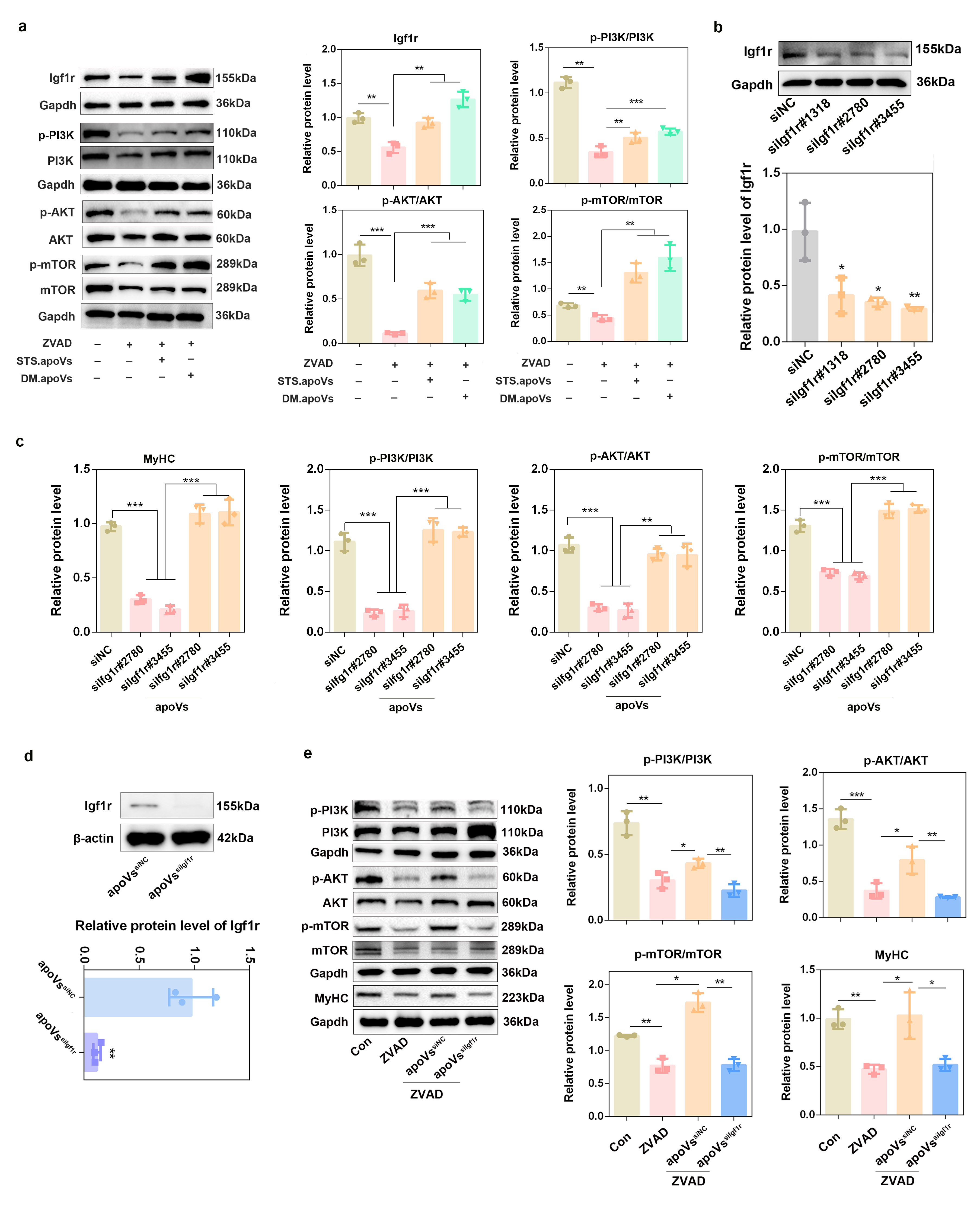


**Fig. S6. C2C12-derived apoVs rescue the impaired myogenic differentiation induced by ZVAD via enhancing Igf1r/PI3K/AKT/mTOR pathway. (a)** STS.apoVs or DM.apoVs were used to treat ZVAD pre-treated C2C12 myoblasts. The protein level of Igf1r, PI3K, AKT, p-AKT, mTOR and p-mTOR was detected by western blot analysis after 3 days of differentiation. Gapdh was used as the loading control, and protein signal intensities were analyzed using ImageJ software (n = 3). **(b)** Igf1r knockdown efficiency in C2C12 cells was determined by western blot analysis. Gapdh was used as the loading control. Protein signal intensities were analyzed using ImageJ software (n = 3). **(c)** Data analysis for the MyHC expression and the ration of p-PI3K/PI3K, p-AKT/AKT and p-mTOR/mTOR after C2C12 cells were co-treated with siIgf1r andapoVs. **(d)** Igf1r knockdown efficiency in apoVs was determined by western blot analysis. β-actin was used as the loading control for C2C12-derived apoVs. Protein signal intensities were analyzed using ImageJ software (n = 3). **(e)** ZVAD pre-treated C2C12 myoblasts were treated with apoVs^siNC^or apoVs^siIgf1r^. After 3 days of differentiation, the protein level of p-PI3K, PI3K, AKT, p-AKT, mTOR and p-mTOR was detected by western blot analysis. Gapdh was used as the loading control, and protein signal intensities were analyzed using ImageJ software (n = 3). **P* < 0.05, ***P* < 0.01, ****P* < 0.001, ns, not significant


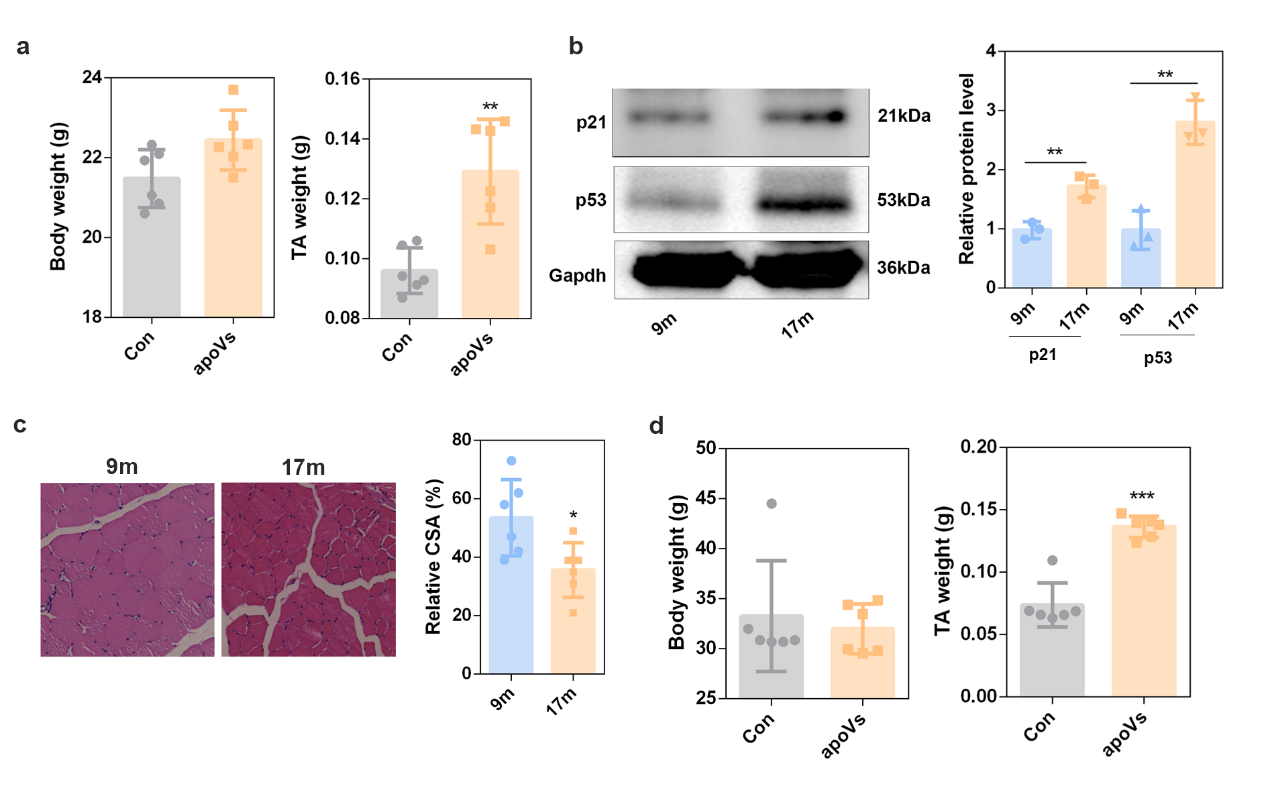


**Fig. S7. ApoVs injection increases the weight of skeletal muscle. (a)** The body weight and TA muscle weight was assessed in apoVs-injected 8-week-old mice (n = 6). **(b)** The protein level of p21 and p53 was detected by western blot for 9-month-old and 17-month-old mice. Gapdh was used as the loading control, and protein signal intensities were analyzed using ImageJ software (n = 3). **(c)** H&E staining images of TA muscles isolated from 9-month-old and 17-month-old mice, and the relative area was assessed using ImageJ software (n = 6). **(d)** The body weight and TA muscle weight was assessed in apoVs-injected aged mice (n = 6). **P* < 0.05, ***P* < 0.01, ****P* < 0.001
